# Highly Customizable 3D Microelectrode Arrays for In Vitro and In Vivo Neuronal Tissue Recordings

**DOI:** 10.1002/advs.202305944

**Published:** 2024-01-19

**Authors:** J. Abu Shihada, M. Jung, S. Decke, L. Koschinski, S. Musall, V. Rincón Montes, A. Offenhäusser

**Affiliations:** ^1^ Institute of Biological Information Processing (IBI‐3) – Bioelectronics Forschungszentrum 52428 Jülich Germany; ^2^ RWTH Aachen University 52062 Aachen Germany; ^3^ Helmholtz Nano Facility (HNF) Forschungszentrum Jülich 52428 Jülich Germany; ^4^ Faculty of Medicine Institute of Experimental Epileptology and Cognition Research University of Bonn 53127 Bonn Germany; ^5^ University Hospital Bonn 53127 Bonn Germany

**Keywords:** 3D flexible implants, 3D microelectrode arrays, 3D printing, neural interfaces, two‐photon polymerization

## Abstract

Planar microelectrode arrays (MEAs) for – in vitro or in vivo – neuronal signal recordings lack the spatial resolution and sufficient signal‐to‐noise ratio (SNR) required for a detailed understanding of neural network function and synaptic plasticity. To overcome these limitations, a highly customizable three‐dimensional (3D) printing process is used in combination with thin film technology and a self‐aligned template‐assisted electrochemical deposition process to fabricate 3D‐printed‐based MEAs on stiff or flexible substrates. Devices with design flexibility and physical robustness are shown for recording neural activity in different in vitro and in vivo applications, achieving high‐aspect ratio 3D microelectrodes of up to 33:1. Here, MEAs successfully record neural activity in 3D neuronal cultures, retinal explants, and the cortex of living mice, thereby demonstrating the versatility of the 3D MEA while maintaining high‐quality neural recordings. Customizable 3D MEAs provide unique opportunities to study neural activity under regular or various pathological conditions, both in vitro and in vivo, and contribute to the development of drug screening and neuromodulation systems that can accurately monitor the activity of large neural networks over time.

## Graphical abstract

Two‐photon polymerization (2PP) lithography and surface micromachining technology allow the rapid fabrication of polymer templates to guide the growth of electrodes for three‐dimensional microelectrode arrays (3D MEAs), enabling in turn, device customizability, high signal‐to‐noise ratio (SNR) recordings, low bending stiffness, and minimized cross‐sectional footprint for in vitro and in vivo neural applications: from 3D neuronal cell cultures to the cortex of living mice.

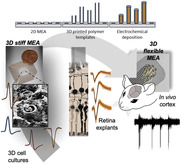

## 1 Introduction

Understanding neural function under regular and pathological conditions requires monitoring neural activity from complex, interconnected networks with high spatiotemporal resolution and over extended time periods. To date, microelectrode arrays (MEAs) are the gold standard for extracellular neural recording and modulation. MEAs comprising a two‐dimensional (2D) spatial arrangement of planar electrodes (hereafter referred to as planar MEAs) are usually used to simultaneously monitor neural activity at multiple sites in 2D systems (e.g., 2D cell cultures). In some cases, planar MEAs also hold nanostructures on the electrodes (e.g., mushrooms or pillars) to enhance the adhesion and electrical coupling of biological cells. Nonetheless, planar MEAs only provide limited access to neural activity in more complex three‐dimensional (3D) neural tissues,^[^ ^1^ ^]^ such as brain slices or organoids.^[^ ^2^ ^]^ First, planar MEAs cannot access the inner volume of such biological systems although the 3D intraneural space is often comprised of multilayered neural structures with distinct cell types and densities that are crucial for neural network function. Moreover, the electrical coupling, and therefore the recording quality, of planar MEAs is often not high enough for the long‐term recording and investigation of active neural networks, such as cultured brain slices. This is in part because the tissue surface often contains dead cells from the slicing procedure that can create a physical barrier between the planar MEA and the living neurons, reducing the signal‐to‐noise ratio (SNR) during neural recordings and increasing the current thresholds for effective electrical stimulation.^[^ ^1^, ^3^ ^]^

Most of the above challenges can be addressed with penetrating MEAs, which are designed for recording and stimulation purposes of intraneural tissue. Penetrating MEAs consist of insulated shafts containing either an active sensing region at their tip (“Utah arrays”) or multiple electrode sites along the shaft (“Michigan arrays”).^[^ ^4^, ^5^, ^6^, ^7^ ^]^ The Utah^[^ ^4^ ^]^ and Michigan^[^ ^7^ ^]^ probe types have been most widely used as the gold standard architecture and form the basis for the transformative field of invasive brain‐computer interfaces (BCIs) and have been proposed for organoid electrophysiology.^[^ ^8^, ^9^, ^10^ ^]^ The sampling capability, a function of both electrode density and the ability to optimally target brain regions of interest, is the primary determinant for their successful use in intracortical BCIs and in primary research on cognitive processes in the human brain.^[^ ^11^ ^]^ Current fabrication methods have made significant advances in recording density as with the silicon (Si)‐based Neuropixels probe^[^ ^12^ ^]^ using complementary metal oxide semiconductor (CMOS) technology. However, failure of such Si‐based electrodes is frequently caused by connector and material problems associated with their stiffness.^[^ ^13^ ^]^

Over the past thirty years, Utah arrays have been very successful in recording neural activity from deep brain regions. Recent Si technology advances featuring the fabrication of Utah‐like arrays have allowed design flexibility and processing capabilities for the implementation of distinct electrode layouts, high‐density electrodes, distinct needle geometries, and arbitrary needle heights, thereby allowing either a 2D (all shafts with the same height) or a 3D (e.g., Utah slant array) electrode spatial arrangement of penetrating MEAs. Nonetheless, the manufacturing processing load and complexity is still high, as multiple dry and wet etchings steps, as well as wafer bonding techniques are needed. Additionally, further cross‐sectional optimization towards reduced bending stiffness to diminish foreign body reactions in chronic applications is still a challenge.^[^ ^4^, ^14^, ^15^ ^]^

Another approach to form MEAs that allow access to the 3D space of neural tissues is to stack multiple densely packed in‐plane Michigan‐style probes with 400–700 µm spacing. However, the pitch between stacked shanks is limited by the dimensions of the spacers required for assembly and bonding.^[^ ^16^, ^17^ ^]^Recently, bundled microwire arrays have been used to massively scale the number of recording channels by integrating with high‐resolution CMOS sensing circuits.^[^ ^18^ ^]^ Alternatively, as an attempt to reduce the mechanical mismatch between the penetrating MEA and the target biological host, ultrathin flexible polymer‐based threads containing multiple electrodes are used.^[^ ^19^, ^20^, ^21^ ^]^ Such probes can reliably record brain activity across the depths of the cortex and cause minimal scarring within the cortical tissue^[^ ^19^, ^20^ ^]^ but require advanced robotic implantation devices when aiming for larger arrays.^[^ ^21^ ^]^

To simplify the spatial arrangement of penetrating MEAs, hereafter, 3D MEAs will be referring to MEAs containing multiple protruding microelectrodes or penetrating shafts. Hence, various types of 3D MEAs for interfacing neuronal tissue have been fabricated using microelectronics fabrication techniques, which primarily involve the deposition or growth of electrode material (e.g., metal, carbon, ceramic, etc.) on the top or bottom surface of substrates.^[^ ^22^, ^23^ ^]^ In recent years, MEA fabrication methods expanded to also include the fabrication of soft MEAs directly on polydimethylsiloxane (PDMS), agarose, and gelatin substrates using inkjet printing as a patterning tool, which also allows for a rapid prototyping approach.^[^ ^24^ ^]^ In addition, inkjet printing has been used to enable the fabrication of 3D microelectrodes on planar MEA substrates.^[^ ^25^ ^]^ By printing nanoparticles, high‐density 3D MEAs with arbitrary variations in shaft height, diameter, and routing were developed and applied in in vivo experiments.^[^ ^26^ ^]^ Moreover, stereolithography 3D printing has been used in combination with ink casting and electroplating of metal electrodes for the fabrication of 3D MEAs in well plates.^[^ ^27^ ^]^ Nonetheless, these approaches have a limited printing resolution down to tens of micrometers, require conductive inks with a high Young's modulus that leads to a higher cross‐sectional footprint, and either lack or require an additional step to implement an insulating layer to passivate the printed electrodes.

Two‐Photon Polymerization (2PP) is one of the most versatile and precise additive manufacturing processes, enabling the production of arbitrary 3D prototypes directly from computer‐aided design (CAD) models with resolutions down to 100 nm. This method is an ideal candidate for biological applications since structures of arbitrary geometry on the size scale of individual cells or even sub‐cellular structures can be reliably reproduced.^[^ ^28^ ^]^ A wide variety of micro pillar arrays, geometries, and materials have been fabricated using 2PP, demonstrating the robustness and broad applicability of the approach.^[^ ^29^ ^]^ 3D laser lithography based on 2PP has several advantages over other conventional microfabrication techniques, including the fabrication of structures with superior resolution (around tens of nanometers) and high aspect ratio (*e.g*., 10:1) on a large variety of substrates. In addition, commercially available photoresins are biocompatible and non‐cytotoxic and have a Young's modulus in the GPa range. Current 3D MEA fabrication approaches based on 2PP technology consist of 3D printed pillars with needle‐like structures that are sputtered with a thin metal stack and passivated with a thin film polymer that is then etched at the electrode tips with laser ablation.^[^ ^30^, ^31^ ^]^ The latter requires a femtosecond laser that etches one probe at a time, as well as intricate optimizations to etch the desired depths and widths with precise µm‐resolution.^[^ ^30^ ^]^

Here, we present a novel and highly customizable array for the fabrication of 3D MEAs by combining 2PP printing with thin film technology. In contrast to state‐of‐the‐art 3D MEA systems, our approach enables fast and reliable processing by 3D printing pillars on planar MEA substrates, whose design and materials are freely selectable. The pillars function as templates to grow metal electrodes in the third dimension while serving at the same time as a passivation layer. Additionally, exploring the limits of the fabrication process, we reduced the form factor of our electrodes to minimize tissue damage when inserting them into neural tissue. Hence, our approach enables the fabrication of custom MEA designs, spanning from stiff 3D MEAs to flexible implants that better match the anatomy and the mechanical properties of the target neural tissue for in vitro and in vivo applications. We show the fabrication of 3D pillar microelectrodes with diameters below 8 µm and lengths of hundreds of µm, demonstrating also strong neural recording capabilities and mechanical stability for the functional assessment of complex population activity patterns that are generated from electrogenic cells within different structurally complex 3D neural tissues.

## 2 Results and Discussion

### 2.1 Fabrication of Highly Customizable 3D Microelectrodes for Neuronal Recordings

**Figure 1:**
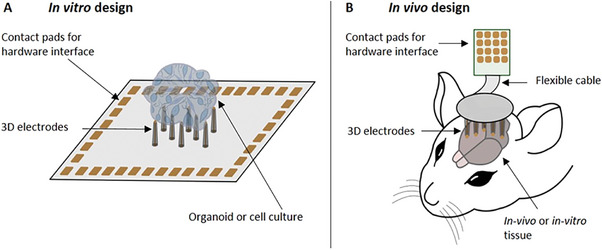
Application modalities of 3D MEAs. A) The in vitro design typically integrates both the culture dish and the 3D MEA directly on the chip, representing a stand‐alone device that can be used for spheroids, organoids, and acute neural slices. B) The in vivo design decouples the MEA from the neuronal tissue, where the MEA is fabricated separately on a (flexible) substrate and flip‐chip bonded to a printed circuit board. Attached to a micromanipulator, the probe can be lowered to penetrate the neuronal tissue in in vitro and in vivo experiments.

We developed highly customizable 3D MEAs for two complementary application modalities to allow neuronal recordings for in vitro (**Figure** **1**A) and in vivo (Figure 1B) approaches. For the in vitro approach, 3D pillar electrodes are directly integrated on chips and retain traditional features of planar MEAs, such as easy hardware integration. The chip represents a stand‐alone device that can be used for electrophysiological recordings of spheroids, organoids, and acute neural slices. In contrast, the in vivo approach is inspired by the deployment of implantable neural probes, where the 3D MEAs are inserted into neural tissue. Due to its flexible design, the in vivo approach can be also used for in vitro applications targeting 3D neural tissues.

**Figure 2:**
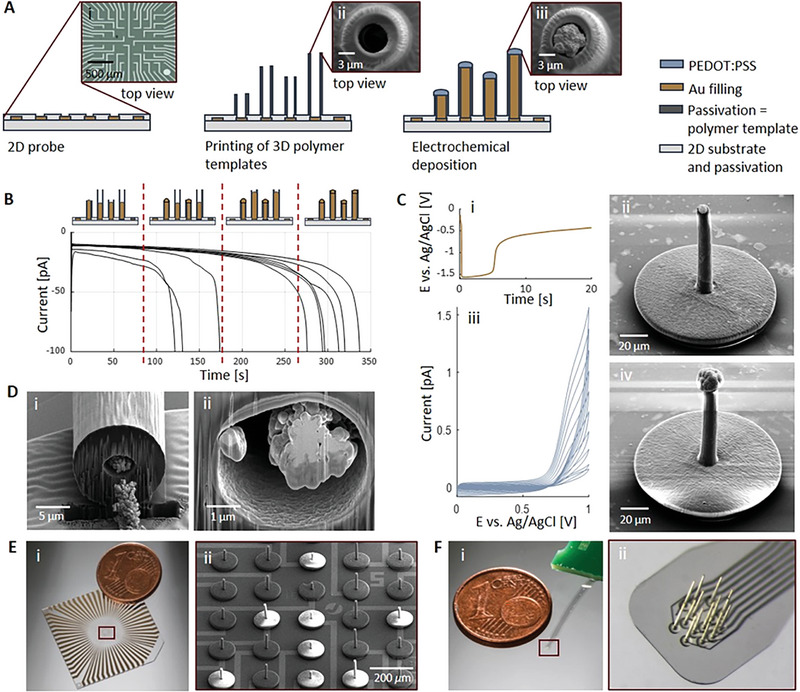
Fabrication of 3D MEAs. A) Fabrication of 3D electrodes on a planar MEA substrate (A_i_) by printing 3D polymer templates (A_ii_) and the template‐assisted electrodeposition of Au and PEDOT:PSS. By carefully controlling the current, a cap can be formed at the top of the pillar (A_iii_). B) Template‐assisted electrochemical deposition of Au inside straight pillars of 35 and 65 µm height. During the chronoamperometry process a constant potential of −1.3 V was used. The current‐time curve exhibits the four stages of an electrochemical deposition process to fill pillars with different heights with Au, which was stopped when the measured current began to increase exponentially, thereby indicating that the Au filling reached the top end of the pillar. C) During the second deposition step, the current was fixed to −100 nA for 20 s (C_i_) to create a smooth and small Au cap (C_ii_). The following PEDOT:PSS deposition was carried out via cyclic voltammetry in 2–10 cycles (in (C_iii_) number of cycles was 10) depending on the desired size of the PEDOT:PSS cap (C_iv_). D) Focused ion beam (FIB)‐cuts of one individual pillar at the base, revealing a wall‐thickness of ≈4 µm (D_i_) and top end (D_ii_) of the pillar. E) Fabrication results showing a stiff 3D MEA device (E_i_) and a zoom in picture of an array with 3D printed pillars of different heights (40–100 µm) (E_ii_)). F) Fabrication results showing a flexible 3D MEA (F_i_) and a zoom in picture of the 3D printed pillars with a height of 500 µm (F_ii_).

As depicted in **Figure** **2**A, the fabrication of 3D MEAs consisted of only three main steps: fabrication of a planar MEA, printing of 3D hollow polymer pillars, and the electrochemical deposition of a conductive material. First, rigid, or flexible planar MEAs (Figure 2A_i_) were fabricated by conventional (or maskless) photolithography processes. The size, pitch, and arrangement of the microelectrodes were then adapted to the different experimental requirements. For the rigid planar MEAs, the conductive titanium/gold/titanium (Ti/Au/Ti)‐layer that forms the microelectrode, interconnects, and contact pads is deposited on an insulating substrate (silica or glass) and covered with SU‐8 for insulation. For the flexible planar MEAs, the conductive Ti/Au/Ti‐layer is embedded between two parylene‐C (PaC) layers (5 µm thick each). For both MEA types, the top passivation and the Ti layer were etched at the contact pads and microelectrode openings, thereby exposing the Au surface. In the next step (Figure 2A_ii_), hollow polymer pillars were printed at the electrode sites of the rigid or flexible planar MEAs using 2PP and a biocompatible^[^ ^32^, ^33^, ^34^ ^]^ photoresin, such as IP‐L (Nanoscribe GmbH & Co KG, Eggenstein‐Leopoldshafen, Germany). To enhance the adhesion between the 2PP‐based structures and the planar MEA, the contact area was enlarged by adding a 3 µm‐thick doughnut‐shaped polymer disk (also referred to as base plate) to the base of the hollow pillar. The opening of the base plate‐pillar element was then aligned to the microelectrode and printed on top of the planar MEA, printing both seamlessly as one coherent element. Hence, by adding a base plate of 50–100 µm in diameter or in the case of narrower pitches, a continuous base plate matching the printing area window, the contact area of the pillar increases by 30.5–124‐fold (see Experimental Section). Lastly, the polymer pillars, serving as templates and a passivation layer at the same time, were filled with Au via electrochemical deposition (Figure 2A_iii_), followed by the deposition of an electrode coating cap by the electropolymerization of poly(3,4‐ethylenedioxythiophene):polystyrene sulfonate (PEDOT:PSS).

In the 2PP printing process (Figure 2A_ii_), the mechanical stability of the pillars is influenced by the microfabrication process, the mechanical properties of the photoresin, the geometry of the pillar, and the pillar pitch within an array. After 2PP exposure, the uncrosslinked polymer is removed when immersed into an organic solvent (development step), such as isopropanol (IPA), and the crosslinked polymer stays forming the print. In such a process, the structures are subjected to capillary forces (*F_C_*) caused by the surface tension of the organic solvents and by the elastic restoring forces (*F_E_*) of the pillars. The latter describes the resistance of the pillars to *F_C_*. While *F_C_* is inversely proportional to the spacing between the pillars, *F_E_* increases with higher Young's moduli and higher pillar diameters and decreases with increasing pillar heights.^[^ ^35^ ^]^ Thus, the closer the pillars are printed next to each other, the higher is *F_C_* and the lower is *F_E._* If *F_C_* is higher than *F_E_*, the pillars will bend towards each other. Therefore, to explore the limitations of the technology, we evaluated different design parameters (**Table** **1**).

**Table 1:** Customizable features and tested limitations of 3D MEAs.

Feature	Explored configurations and tested values	
Substrate materials	Parylene‐C and SU‐8 on quartz	
Pillar material	IP‐L	
Electrode material	Au, PEDOT:PSS	
Pillar height	Up to 500 µm straight pillars with 12 µm outer diameter and 200 µm pitch	
Pillar diameter and wall thickness	Down to 12/8 µm outer/inner diameter (2 µm wall thickness) for straight pillars of 500 µm height	
Pillar pitch and height	The pitch highly depends on the pillar geometry and height:	
	Pitch [µm]	Maximal possible height [µm]
	20	50
	25	150
	35	200
	200	500
Electrochemical deposition	Au‐filled pillars of up to 400 µm height with an inner diameter of 8 µm	
Pillar electrode arrangement	Grid (e.g., Figure 4), multisite (e.g., Figure S5, Supporting Information)	
3D MEA application modality	in vitro (standalone device), in vivo (implantable device)	

Firstly, we tested the maximum pillar height possible with the thinnest wall thickness possible. To this end, we varied the height, as well as the outer and inner pillar diameters (Figure S1A,B, Supporting Information), which yielded a stable 2PP printing process for pillars as high as 500 µm with a wall thickness of 2 µm (outer/inner diameters as small as 12/8 µm). Additionally, when designing and fabricating arrays of pillars, the pillar pitch in the MEA design is an important parameter (see above). To assess the minimum pitch with the maximum pillar heights possible, we then printed pillar arrays with varying pitches from 20–200 µm and heights up to 500 µm, achieving stable pillar arrays with a minimum pitch of 20, 25, 35, and 200 µm for pillar heights of 50, 150, 200, and 500 µm, respectively (Figure S1C–E, Supporting Information).

While we achieved high aspect ratios of up to 500:12 after 2PP printing, future applications, such as targeting deeper cortical regions, the use of bigger animal models, human‐based organoids, or even human applications, require the implementation of even longer pillars (> 500 µm). The current processing leads to pillars with a low bending stiffness (down to 3.86 N µm^2^, Table S1, Supporting Information), due to the Young's modulus of the polymer and the high aspect ratios of our designs. Such characteristics are advantageous for implantable devices but can generate mechanical instability in longer pillars during manufacturing, as the *F_C_* surpasses *F_E_* causing the collapse of the free‐end pillars due to bending and merging them during the drying process after development. Thus, to further enhance mechanical stability during 2PP processing, an additional UV‐exposure while the structures are immersed into isopropanol can be implemented as proposed by Purtov et al.^[^ ^35^ ^]^ Such a process can strengthen the crosslinking density of the polymer, which increases, in turn, the Young's modulus of the polymer, and therefore enhances the mechanical stability of the pillars during fabrication.^[^ ^35^ ^]^

2PP lithography is a high‐precision additive manufacturing process that can be used as a prototyping and small‐volume manufacturing tool. However, such a fabrication process is not yet scalable to the mass‐production capabilities of contact lithography. Advancements in optics (e.g., an objective lens with a bigger focal volume), laser technology (e.g., higher laser power), and the development of new 2PP photoresins are still needed for the scalability of the process.^[^ ^36^ ^]^ Nonetheless, the process can still be used to customize planar MEA substrates with 3D electrodes to suffice electrophysiological niche requirements. To reduce printing time, design optimization is possible if multiple 3D pillars with a total height of up to 300 µm fit in a printing area of 285×285 µm^2^ (e.g., 2–4 pillars if the base plate is 100 or 50 µm in diameter). For bigger design dimensions, block splitting is needed during the 2PP process. Currently, our 2PP printing time follows a power fit (see Figure S2, Supporting Information), indicating that approximately 38.4 min are required to modify a planar MEA holding 256 microelectrodes with 256 hollow pillars, each with an aspect ratio of 5.83.

While the methodology exposed here is based on 2PP lithography for the fabrication of self‐aligned polymer‐based templates that guide the electrodeposition of Au and passivate the conductive electrode at the same time, it is also possible to use standard contact photolithography processes to create the self‐aligned polymer templates by sacrificing design customizability. For example, by using thick polymers, such as SU‐8 or polyimide. However, the process will be limited by the achievable thickness of the polymeric layers (usually up to 200 µm), indicating that multiple layers would be needed, and etch‐back processes or bonding techniques need to be established to implement thick polymeric layers that will define the height of the pillar templates.

Furthermore, to ensure a successful electrochemical deposition process (Figure 2A_iii_), the base plate printed to enhance the adhesion and prevent leaks for the Au solution between the pillars and the planar MEA was crucial to prevent failure due to the overgrowth of Au at the bottom end of the pillars (Figure S3, Supporting Information). Thereupon, the template‐assisted electrodeposition process of Au was carried out in two steps, using first a constant potential for a fast and rough Au growth (Figure 2B) and then a fixed current (Figure 2C_i_) to ensure the remaining filling of the template with a smoother morphology (Figure 2A_iii_,C_ii_). Likewise, the Au‐filling within the pillar exposed by focused ion beam (FIB)‐cuts shows a string (Figure 2D) with a dendrite‐like Au structure at the base of the pillar (Figure 2D_i_) and a smoother but still rough Au morphology at the top end of the pillar that creates a Au cap (Figure 2A_iii_,C_ii_). It was therefore beneficial to perform the electrodeposition in different steps to ensure the desired surface morphology during Au growth.

Hence, with the proposed methodology (for details, see Experimental Section) it was possible to fill pillars up to 400 µm in height and 12/8 µm outer/inner diameter (see fabrication limits in Table 1 and Figure S1F, Supporting Information), which was sufficient for the required applications of this work (see Section 2.3). Nonetheless, filling up taller pillars is still a challenge as the process is limited by ion diffusion and ion charge transfer mechanisms that define the morphology of the deposited Au. Therefore, to further optimize the electrodeposition of Au, strategies such as heating the Au solution or using higher ion concentrations to enhance the velocity of the process can be adopted.

Additionally, to improve the electrochemical performance and increase the electrode surface area that would be in contact with the neural targets, a PEDOT:PSS electrode coating was deposited via cyclic voltammetry (CV) as the last deposition step (Figure 2C_iii_). Such a process allowed the formation of a cap‐like structure on top of the previously electrodeposited Au (Figure 2C_iv_), creating a 3D structure that enables a tight contact between electrogenic cells and the electrode, similar to 3D mushroom‐like structures described in the literature.^[^ ^28^, ^29^ ^]^ Depending on the number of CV cycles, the size of the resulting PEDOT:PSS cap can be precisely controlled and adjusted to specific needs (Figure S4, Supporting Information). For instance, Figure 2C_iv_ shows a PEDOT:PSS cap with a diameter of 20 µm after ten CV cycles. The surface morphology of the electrodeposited PEDOT:PSS is corrugated and rounded, but can also be cauliflower‐like (Figure S4, Supporting Information), as it depends on the underlying Au structure.^[^ ^37^ ^]^

Hence, our technological approach allows the fabrication of 3D printed pillar electrodes with high aspect ratios of up to 33:1 and electrode diameters down to 8 µm (Table 1; Figure S1, Supporting Information), thereby surpassing aspect ratios of up to 11:1 and electrode diameters down to 10 µm, as reported in the literature when using 2PP printing processes^[^ ^30^, ^31^ ^]^The proposed technology allows the fabrication of hollow polymer pillars that are custom‐built and that can be individually addressable (e.g., during electrodeposition), therefore offering maximum flexibility to meet the requirements of different use cases. Given the customization available with 2PP, any desired pillar shape is only limited by high aspect ratios defined by the ratio between the height and the outer diameter of the pillar template and the pillar pitch. Hence, pillars with different heights on the same MEA can be printed (Figure 2E_ii_; Figure S1B, Supporting Information) and multisite pillar designs (Figure S5, Supporting Information) can be implemented without extra effort with the purpose of recording from different neural layers at the same time (see Section 2.3), therefore enabling a 3D spatial arrangement of microelectrodes in our MEA devices. Furthermore, the pillars exhibited higher stability as theoretically expected, given that the wall thickness of the hollow pillars at the base of the pillar was 4 µm instead of 2 µm, as revealed by FIB‐cut analysis (Figure 2D_i_).

Finally, we also explored the possibility of printing on different substrates and found that printing on transparent polymers such as PaC is feasible. Thus, we were able to fabricate 3D electrodes on top of rigid planar glass MEAs for in vitro applications (Figure 2E), as well as on flexible PaC substrates for in vivo applications (Figure 2F). Successful fabrication of 3D MEAs is then determined by five main processing characteristics: identification of the printing interface, the proper development of uncrosslinked 2PP photoresin, the alignment of the print to the planar MEA, the shelf life of the 2PP photoresin and the electrodeposition solutions, and the environmental conditions at which the process is performed.

During the 2PP process, finding the substrate‐photoresin interface is of high importance, as it determines the starting focal point of the print. An incorrect printing interface can affect the end height of the print, for example, shorter pillars, or lead to adhesion problems. Moreover, underdeveloped pillars and misalignment of the print are sources of errors that can prevent the Au salt solution from going inside the pillar and contacting the microelectrode on the planar MEA. Additionally, the optical stability of the 2PP photoresin, determined by the shelf life, and stable environmental conditions can lead to changes in the printing parameters, such as adjustment of laser power and scan speed.

Given the above, a yield of 85% was achieved in the 2PP printing process and 55% after the electrodeposition process. To further increase the reproducibility of the process, alignment markers made from for example Au, which has a low refractive index, can be implemented to automate the interface finding and the alignment of the print on the planar MEA. Hence, given that the process exposed here allows customization of materials and design of the 3D MEA (Table 1), our technology meets the needs of different use cases. Stand‐alone planar rigid MEAs with easy hardware integration to allow the growth of cell cultures, organoids, and acute neural slices, and flexible MEAs to enable implantable neuroelectronic applications and enhance the integration of the device with soft neural tissue.

### 2.2 Characterization of 3D MEAs

#### 2.2.1 Impedance Spectroscopy after Au and PEDOT:PSS Deposition

**Figure 3:**
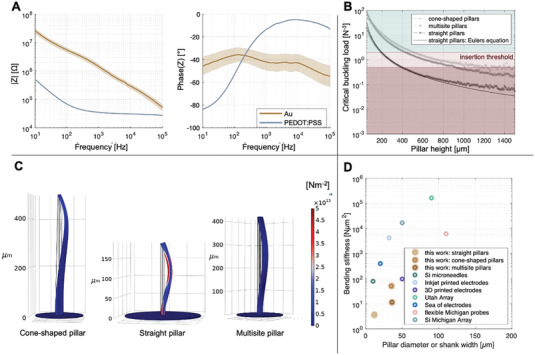
Characterization of 3D printed pillar electrodes. A) Bode plot of electrochemical impedance spectroscopy measurements of an example probe with 12 electrodes after Au (gold) and PEDOT:PSS (blue) electrodeposition. Mean values are shown in solid lines and the mean +/‐ standard error mean in shaded area. B) Analytical calculation and COMSOL simulation of the critical buckling load following Euler's equation for a fixed‐pinned boundary condition for cone‐shaped, straight, and multisite pillars of different heights. In light red, an insertion force threshold between 0.5–2 mN is shown. C) COMSOL simulation of Von Misses’ stress showing the highest possible pillars according to the critical buckling load (490 µm for cone‐shaped pillars, 190 µm for straight pillars, and 420 µm for multisite pillars with 3 electrodes). D) Literature comparison of bending stiffness with other common materials and designs for implantable devices (see Table S1, Supporting Information for the references and calculations).

Electrochemical characterization showed that the impedance of the electrodes after the deposition of the PEDOT:PSS electrode coating cap was reduced by 29‐fold. **Figure** **3**A shows the impedance spectra after Au and PEDOT:PSS electrodeposition for typical pillar electrodes with a height of 100 µm and a diameter of 8 µm, exhibiting an impedance of 1.2 MΩ and 35.2 kΩ at 1 kHz after Au and PEDOT:PSS electrodeposition, respectively (Figure 3A). The pillar electrodes therefore exhibited an electrochemical performance that is in range for electrophysiological measurements.^[^ ^38^, ^39^ ^]^

Impedance values of the 3D Au electrodes at 1 kHz are slightly higher compared to standard 2D electrodes with 10 µm diameter (1 MOhm at 1 kHz^[^ ^40^ ^]^). This indicates that the Au does not overgrow the pillar but covers only the 8 µm wide pillar opening. The impedance amplitude decreases with increasing frequency, exhibiting the typical behavior of an Au electrode.^[^ ^40^ ^]^ When depositing PEDOT:PSS onto the Au, the impedance changes to a rather resistive behavior in the frequency range of interest, between 1 kHz and 100 kHz.^[^ ^38^ ^]^ This change can be explained by the additional ionic conductivity of PEDOT:PSS and coincides well with findings in the literature.^[^ ^40^, ^41^ ^]^ Given the low impedance after PEDOT:PSS electrodeposition, the 3D electrodes exhibited a thermal noise of 0.8 µV for a bandwidth of 300 Hz to 3 kHz.

#### 2.2.2 Mechanical Performance of 3D MEAs

The physical dimensions of the pillars are important parameters to assess the mechanical performance of the 3D MEA in implantable neural applications, especially when planning the insertion and assessing the compliance of the 3D probes within the target tissue. To assess the insertion probability of the pillars into neural tissue, we investigated the critical buckling load (*P_e_*) of the pillars, which highly depends on their physical properties and dimensions. According to the literature, an insertion force between 0.5 and 2 mN is needed to penetrate neural tissue such as the brain or the retina.^[^ ^42^ ^]^ If the insertion force is greater than the *P_e_* of the pillar, insertion failure is expected due to bending.^[^ ^42^ ^]^

To determine the best design, we compared straight, cone‐shaped, and multisite pillars. The straight pillars comprised an inner and outer diameter of 8 µm and 12 µm, respectively. The cone‐shaped pillars had an outer diameter of 35 µm and an inner diameter of 30 µm at the base, and an outer diameter of 12 µm and inner diameter of 8 µm at the pillar end. For multisite pillars, we chose three straight pillars next to each other, each one comprising 8 µm inner and 12 µm outer diameter, respectively (Figure S5, Supporting Information). Assuming a fixed‐pinned boundary condition, *P_e_* is highly improved by using cone‐shaped or multisite pillars versus straight pillars (Figure 3B), indicating that pillars up to 500 µm or 430 µm and 200 µm, respectively, can be used for an aidless penetration of neural tissue when assuming a conservative insertion force threshold of 2 mN. Thus, the insertion feasibility was first confirmed with the successful insertion of the pillars in agarose tissue phantoms (Figure S7, Supporting Information and Video S1, Supporting Information). Additionally, simulations show that cone‐shaped and multisite pillars have lower stresses along the z‐axis, therefore also suggesting a higher stability than straight pillars upon penetration of neural tissues (Figure 3C). Moreover, given that the wall thickness at the base of the pillars was found to be 4 µm (Figure 2D), our simulations remained conservative by assuming a 2 µm wall thickness. Notably, a 4‐µm thick wall further increases the *P_e_* by 75%, suggesting that our pillars are even more stable than theoretically expected.

Considering that using 3D penetrating probes is an invasive electrophysiology method for accessing the 3D intraneural space of neural tissues and given its potential use for chronic in vivo applications, we investigated the bending stiffness describing the compliance of the probes. Figure 3D illustrates the effective bending stiffness of the 3D printed pillars (straight, multisite, and cone‐shaped pillars) in comparison to state‐of‐the‐art 3D neural probes. The effective bending stiffness is proportional to the cross‐section of the probe and the Young's modulus of the material, with a lower bending stiffness reducing the mechanical mismatch between the probe and the tissue and therefore promoting compliant tissue integration.^[^ ^20^ ^]^ The comparison with state‐of‐the‐art probes such as Utah or Michigan arrays, and even new approaches with 3D printing technology, shows the advantage of our 3D printed probes. Although the Young's modulus of IP‐L (4.7 GPa according to the manufacturer) is relatively high compared to softer materials such as PaC (2.76 GPa^[^ ^43^ ^]^), the small dimensions of our pillars led to an optimized cross‐sectional footprint that reduces the effective bending stiffness of our probes, thereby standing out when compared to the literature.

### 2.3 in vitro and in vivo Applications of Customizable 3D MEAs

In this section we highlight the deployment suitability of our 3D MEA technology in different structurally complex 3D neuronal models, such as 3D neuronal cell cultures using a scaffold, explanted neural tissues, such as the retina, and in vivo in the mouse cortex. The successful employment of the 3D MEA technology in all these different use cases demonstrates its potential for studying neural network activity, both in vitro and in vivo, in a highly defined way that can be adjusted to the specific spatial structure of the target tissue. This allows a large range of neuroscientific applications, such as the targeted recording of individual neurons in key locations of a 3D neural network, simultaneous acquisition of multiple layers and neuronal cell types in the retina, or the targeted recording across multiple neural columns or different brain regions in the intact brain. Given the flexibility of our approach to implementing different spatial designs, many other applications, such as chronic recordings in developing organoids or invasive BCIs, are also readily within reach.

#### 2.3.1 Recordings from 3D Neuronal Cell Cultures

The fabricated and characterized 3D MEAs can be used to record the neural network activity from 3D neuronal cell cultures in vitro. Therefore, primary cortical embryonic rat neurons were seeded with high‐density (4000 cells mm^−2^) onto the 3D MEA device with 32 electrodes arranged in a diamond shape with distances of 20 µm to 30 µm and a 2 µm SU‐8 passivation. A 3D in vitro network around the 3D electrodes can only be established by the neurons with the aid of a supporting scaffold. Thus, a 3D scaffold design was added to the CAD geometry file of the 3D pillar templates and printed in one printing step to promote a successful 3D network growth.^[^ ^44^ ^]^ Similar as described for the printed pillars, a 3 µm thick base plate was printed around pillars and cages to enhance the adhesion to the planar MEA. In this case, two pillar heights were used, 35 µm and 70 µm, with the height of the scaffold matching the height of the tallest pillars.

**Figure 4:**
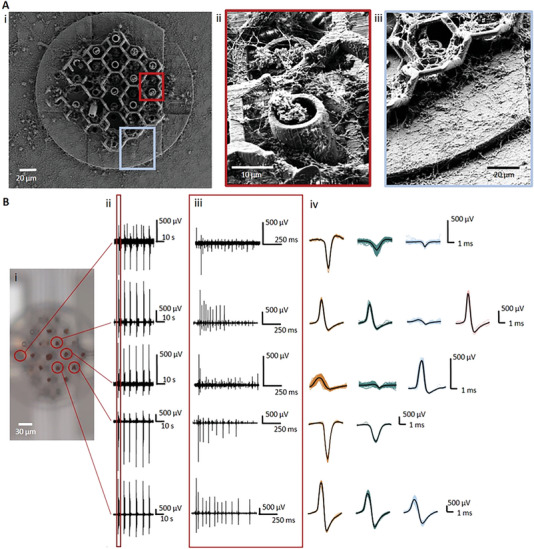
In vitro cell culture with 3D electrodes on planar stiff MEA. A) Scanning electron microscope (SEM) image of a 3D cell‐culture at 14 days in vitro (DIV) of primary cortical embryonic rat neurons within a 3D printed scaffold containing 3D printed pillars that sits on top of the 2D MEA to guide the growth of the neuronal culture. Zoomed in images show that cells do not avoid the 3D prints (ii, red) and cell growth is even enhanced on the scaffold in comparison to the baseplate (iii, blue). B) Electrophysiological recordings from different electrodes (B_i_) showing bursting activity (B_ii_) (zoom‐in (B_iii_)) and different spike shapes (B_iv_) of a 3D cell‐culture of primary cortical embryonic rat neurons at 14 DIV.

To investigate the growth behavior of the neurons inside the 3D scaffold system, the cells were fixed with glutaraldehyde after they established a neural network for 14 days in vitro (DIV). Fixation was followed by a critical point drying (CPD) step that allows for the moderate exchange of the liquid inside the cells with gas without destroying the shape of the cells. **Figure** **4**A shows the scanning electron microscope (SEM) investigation of the established network within the 3D scaffold. Neurons densely populated the 3D printed structures, confirming the biocompatibility of the system. The side‐view of Figure 4A_ii_ also demonstrates the suitability of the scaffold to obtain neural growth in all three dimensions. Neurons which were located within the scaffold at different heights, formed connections with neurons at the bottom using the scaffold system for dendritic and axonal growth support. While the cell bodies did not directly grow on top of the electrodes, the scaffold guided the axons and dendrites towards the electrodes. This can be seen in Figure 4A_iii_ where the cells clearly preferred the scaffold over the base plate. Hence, for in vitro cell recordings, an optimization of the scaffold material, for example using softer materials such as soft silicone rubbers, and the scaffold geometry can lead to enhanced coupling of the neuron‐scaffold interaction, as well as guiding neural growth behavior on the scaffold.

We then performed electrophysiological recordings of the 3D neural networks. Figure 4B shows the recorded signal of five different electrodes with a height of 35 µm or 70 µm, measuring coordinated spontaneous activity in the form of synchronized network bursts after three weeks of culture across the different channels (Figure 4B_ii_). Such behavior is typical in low‐density cultures (700–800 cells mm^−2^) after three weeks, when a mature neuronal network is achieved.^[^ ^45^, ^46^ ^]^ Given the high‐density of our culture, the proximity of nearby neurons promoted network maturation, thereby allowing the electrical behavior of a mature neuronal network after two weeks of culture.^[^ ^47^ ^]^

Moreover, conversely to planar MEAs, where electrical signals are usually hundreds of µV,^[^ ^46^, ^48^ ^]^ the measured action potentials had exceptionally high peak‐to‐peak amplitudes of up to 3.6 mV (Figure 4B; Figure S6, Supporting Information) and a mean SNR of 11.8 +/‐ 5.6, with a maximum SNR of up to 179.7. While 74% of the detected spikes exhibited a spike amplitude below 100 µV, only 3% were higher than 1 mV. The reason for these high‐quality recordings is the improved coupling of the neurons around the 3D Au electrode caps and matches the tight coupling between neurons and other nano‐structured electrodes, such as high aspect ratio nano straws^[^ ^49^ ^]^ and mushroom‐like Au electrodes.^[^ ^50^ ^]^ Furthermore, the characteristic shape of action potential signals ranged from bi‐phasic to monophasic characteristics (Figure 4B_iii_,B_iv_), confirming the recording of multiple units per single electrode. Hence, given the high‐density of the culture, single 3D electrodes could be recording not only from somas but neurites from neighbor neurons.

#### 2.3.2 3D in vitro Recordings from Explanted Rat Retinas

**Figure 5:**
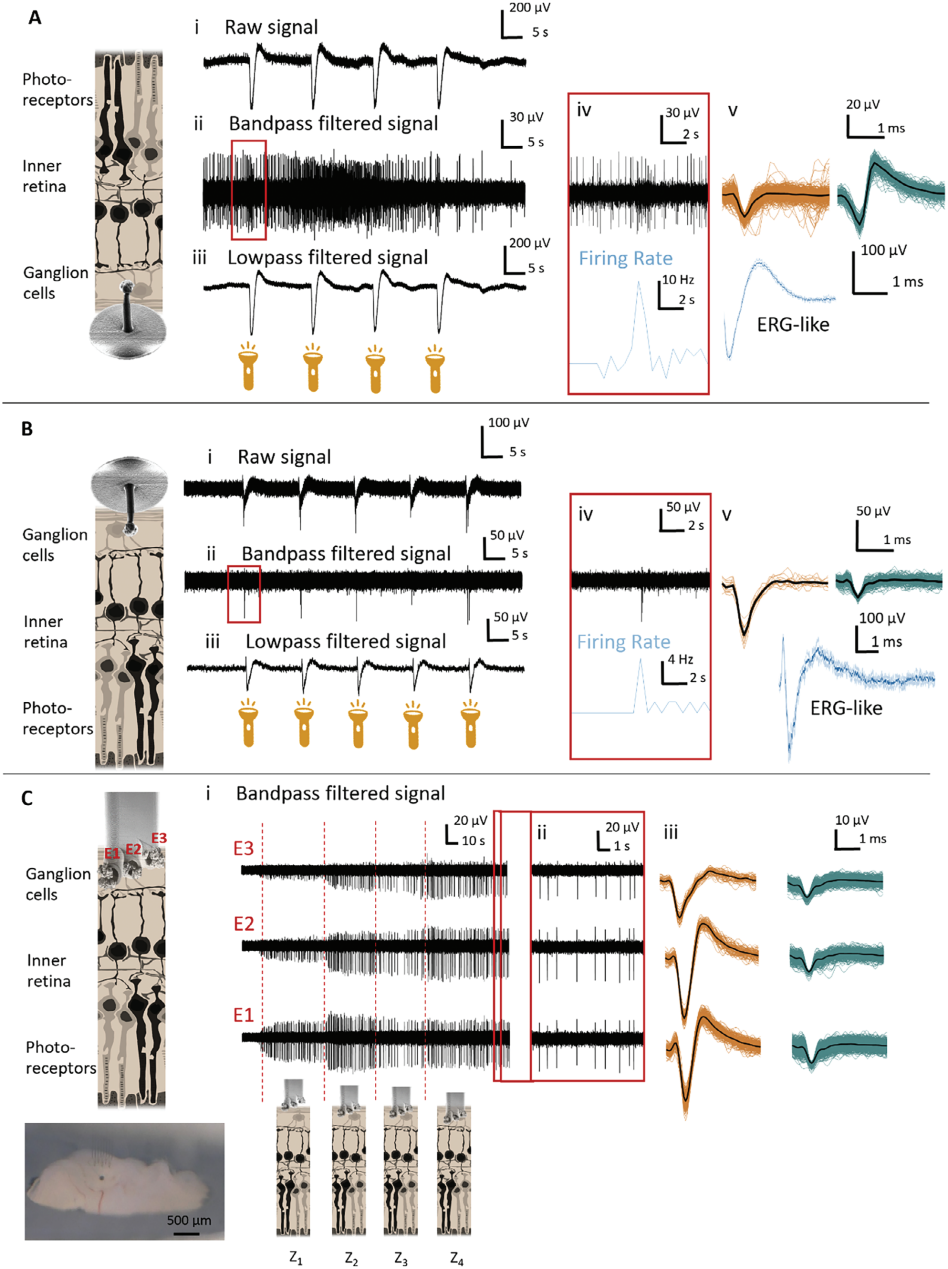
In vitro recordings of explanted rodent retinas. Retinal recordings using in vitro A) and in vivo B) approaches, exhibiting raw electrical signals (A‐B_i_), spiking activity (bandpass filtered signal) (A‐B_ii_), and local field potentials (lowpass filtered signal) (A‐B_iii_) captured upon optical stimulation. Snapshots of individual optical responses in A and B reveal that the firing rate (blue trace) of the spiking signal (black trace) increases upon optical stimulation (A‐B_iv_). In (A‐B_v_) distinct neural waveforms are shown. Additionally, in both cases, averaged ERG‐like waveforms (blue) upon four to five stimuli representing the summed activity of the retina were recorded. C) Insertion of multisite pillars containing three electrodes with a height difference of 20 µm each. Recordings exhibiting the intraretinal placement of a multisite pillar following a stepwise insertion (Z_1_–Z_4_). Additionally, recording extracts at Z_4_ (C_ii_, red window) and spike waveforms captured by individual electrodes (C_iii_) within a single multisite pillar are shown.

To further investigate the application possibilities of our 3D MEAs, we used the in vitro (**Figure** **5**A) and in vivo approaches (Figure 5B,C) to perform electrophysiological recordings from explanted wildtype rat retinas. For the in vitro approach, we used 35‐µm and 70‐µm long individual pillars on a rigid MEA substrate. Whereas for the in vivo approach, we chose 65‐µm long individual pillars (Figure 5B) and multisite pillars with heights of 80, 100 and 120 µm printed directly next to each other (Figure 5C). The rat retina has a thickness of 192 µm^[^ ^51^ ^]^ and placing the electrodes from the epiretinal side, we aimed to reach the ganglion cell layer for electrophysiological recordings. Thus, the dimensions of the 3D MEAs were chosen according to the thickness of the retinal layers and considering the possibility of tissue dimpling and an uneven retinal surface.

In both approaches, we observed clear physiological responses when the retina explant was stimulated with light. Characteristically, local field potentials (LFPs) and an enhanced firing rate of the spiking activity of retinal ganglion cells were time‐locked with optical stimulation (Figure 5A_i‐iv_,B_i‐iv_), as the recording electrodes were most probably placed close to tonic ON‐ganglion cells, which exhibited a sustained spiking response during light stimulation. The LFPs (Figure 5A_iii_,B_iii_ and extracted waveforms in Figure 5A_v_,B_v_) were comparable to reported waveforms of electroretinograms (ERGs) from intraretinal recordings in explanted mouse retinas, which comprise the summed activity of retinal neurons, in specific reflecting the function of photoreceptors and bipolar cells.^[^ ^52^ ^]^ The biphasic waveform of somatic spikes in the retina extracted from the bandpass‐filtered signal (Figure 5A_ii_,B_ii_) were present in the in vitro approach as well as in the in vivo approach, indicating a deep insertion into the retinal ganglion cell layer.^[^ ^53^ ^]^ In the in vitro approach, the observed increased firing rate over the course of the recording (Figure 5A_ii_) is probably due to mechanical stimulation induced by small movements of the retinal tissue that were provoked by the perfusion system in the in vitro setup, as the tissue was not fully fixed onto the surface of the MEA. In both cases, action potentials with peak amplitudes of up to 80 µV, an average SNR of 9.5 +/‐ 6.3, and a maximum SNR of 25.2 were captured.

Considering future applications such as restoring vision through neural stimulation, the in vivo approach is a promising tool for the implementation of a prosthetic device as it could bring the electrodes closer to different neural targets, such as retinal ganglion cells or bipolar cells in the inner retina. Retinas of patients that are affected by retinal degenerative diseases, such as retinitis pigmentosa or age‐related macular degeneration, typically suffer remodeling processes of the retinal network. Moreover, the sensory input due to the photoreceptor‐loss needs to be artificially restored, which may be possible by electrically stimulating the remaining retinal network with the help of prosthetic devices as reported before.^[^ ^54^ ^]^ Thus, as proposed before,^[^ ^52^, ^55^ ^]^ it could be beneficial to adjust the position of the electrodes to different neural targets and explore electrical stimulation protocols that can adjust to the pathologic processes occurring in the diseased retina.

To access the 3D intraretinal space, we therefore implemented and tested a multisite probe (Figure 5C) that comprised three electrodes with 20 µm spacing (heights of 80, 100, and 120 µm), including thereby characteristics of both the Utah and Michigan (multi‐site recordings from one shank) arrays. When recording the electrical activity of retinal ganglion cells during the multisite insertion, we found that the spike amplitude changed with every insertion step. We used a micromanipulator to precisely control the insertion of the implant with an initial step size of 100 µm followed by step sizes of 50 µm with insertion speeds of 185 µm s^−1^. However, considering that the implant itself is flexible and that the retina is a viscoelastic tissue, the step sizes do not directly translate into insertion depths, as the tissue is subjected to dimpling during insertion. Hence, the intraretinal positioning is guided by the electrical activity recorded by the electrodes, as well as the geometry and arrangements of the electrodes in the probe.

Therefore, at the first insertion step (Z1) in Figure 5C, the lowest electrode (E1) was entering the retinal ganglion cell layer (GCL), which contains the spiking ganglion cells, and thus recorded spontaneous activity. The second electrode (E2) picked up lower amplitude spikes as it was further away from the ganglion cells, probably at the retinal surface, and no spiking activity was observed at the third electrode (E3), as its location is still not close enough to pick up the electrophysiological signal. After a second insertion step (Z2), we observed an increase in the spike amplitude in E1 and E2 electrode while E3 also started capturing the spiking activity. As the distance between the electrodes is 20 µm and the GCL is 25–31 µm thick, we assume, that E1 is already located inside the inner plexiform layer (IPL) still picking up spiking activity from the ganglion cell layer, while E2 is placed inside the GCL layer and E3 directly on the retinal surface. When inserting even deeper into the retina (Z3 and Z4), the spiking activity amplitude in E1 decreased whereas the amplitude slightly increased in E2 and E3 which confirms that those electrodes also entered the GCL. Thus, at Z4, E2 was closest to a spiking cell since it shows the highest amplitude spikes.

This recording demonstrates how we can precisely adjust the position of the electrodes within the tissue to optimize neural measurements from different parts of the tissue. When observing individual spikes closer, it becomes clear that we are following the same units in the neural column followed by the three electrodes since the spikes appeared with a small conduction delay of 0.5 to 1 ms in all channels, capturing, in turn, the same waveforms with different amplitudes (zoomed and sorted spikes in Figure 5C_ii_,C_iii_). Such recording behavior matches earlier findings when 2D multisite and penetrating intraretinal probes were employed in the retina.^[^ ^52^, ^55^ ^]^ Accordingly, in this use case, we demonstrated the neural recording capability of tracking the same neuronal column at different depths. Importantly, foreseeing future applications, this methodological approach can allow the identification of the ideal location for both recording and electrical stimulation of neural activity, and would, therefore, be an important feature for future retinal prosthetic devices.

Additionally, we observed mechanical instability of oversized PEDOT:PSS electrode coatings (e.g., after ten CV cycles), which would break at the interface with the Au string inside the pillars upon acute intraretinal insertions. Nonetheless, fragmentation of the PEDOT:PSS electrode coatings was prevented by reducing the size of the conductive polymer cap (e.g., after two CV cycles), yielding robust coatings that showed mechanical and electrochemical stability after multiple insertions and more than 10 times of re‐use (Figure S8A, Supporting Information). Accordingly, the multisite probe demonstrated that even slightly overgrown caps are stable when inserted >10 times. While after multiple insertions the impedance of some of the 3D microelectrodes increased, most PEDOT:PSS caps were still intact and ≈85% of the electrodes maintained suitable electrochemical properties for neural recordings (Figure S8B, Supporting Information). Nonetheless, when not being gentle with the probes while insertion and retraction, the risk of material residues is feasible (Figure S8B_ii_, Supporting Information). In any case, this risk is highly reduced when the formation of PEDOT:PSS caps is precisely controlled during fabrication (Figure S4, Supporting Information). Moreover, given its multiple uses in biological tissue, as expected, retinal tissue residues were encountered in between the electrodes (Figure S8A_ii_, Supporting Information). Therefore, for repetitive uses, the implementation of cleaning protocols must be further investigated.

#### 2.3.3 in vivo Recordings from Mouse Cortex

3D MEA recordings in living animals and humans are a crucial tool to measure the activity of neural populations across the neocortex and are used in various applications, such as invasive BCIs or to study the interaction between cortical areas.^[^ ^56^ ^]^ Our printed 3D MEAs would be an important extension of existing technologies, such as Utah arrays, by allowing custom penetrating electrode designs on flexible materials to target specific combinations of cortical regions or layers of interest. To test the capabilities of our 3D MEAs in vivo, we therefore performed acute recordings, capturing action potentials with peak amplitudes of up to 200 µV and a SNR of up to 31.7 (mean of 6.9 ± 2 standard deviation) in the neocortex of anesthetized mice.

**Figure 6:**
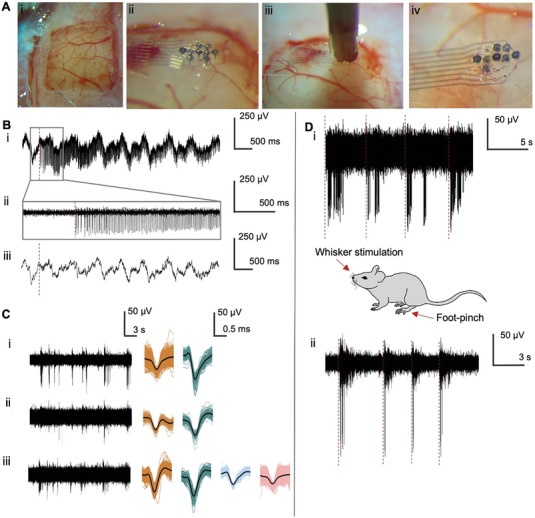
In vivo mouse recording. A) The surgical approach to insert the implant into mouse cortex. After a craniotomy and removing part of the dura (A_i_), the flexible implant was placed on the cortical surface (A_ii_). A wooden rod (A_iii_) was used to push the implant inside the cortex, which subsequently remained in the tissue after retracting the wooden rod (A_iv_). B) Recordings from an example electrode at the time of insertion, showing the raw (B_i_), as well as filtered signals in the high‐ (300 to 3000 Hz, ii) and low‐frequency (up to 300 Hz, iii) range. The red line in (B_i_), (B_ii_), and (B_iii_) denotes immediate spiking activity after insertion whereas the low‐frequency activity was largely unperturbed. C) Example of simultaneous recordings from three electrodes (C_i_), (C_ii_), and (C_iii_) with sorted waveforms from multi‐unit and potential single‐unit recordings. D) Example recording from a pillar electrode with periodic whisker stimulation every 5 s (D_i_) and foot‐pinch stimulation every 3 s (D_ii_).

The probe configuration consisted of a combination of 250 µm long pillars with 100 µm spacing and Au electrodes coated with PEDOT:PSS. After performing a craniotomy and removing the dura (**Figure** **6**A_i_), we used the in vivo approach to insert the electrodes in the primary somatosensory cortex. First, we used a micromanipulator to position the implant on the cortical surface (Figure 6A_ii_). To push the implant into the tissue, we used a blunt wooden rod. The rod was placed directly over the implant, which was then inserted with controlled high‐speed steps of 250 µm with a velocity of 4000 µm s^−1^ (Figure 6A_iii_). The high‐speed steps allowed the pillars to overcome tissue dimpling and penetrate the cortex in a smooth way without creating any visible tissue perturbation (Figure 6A_iv_; Video S2, Supporting Information). Although 3D MEAs are meant to be single use for in vivo applications, the pillars exhibited good adhesion onto the flexible substrate and showed to be mechanically stable during the insertion and retraction of the implant (Videos S2 and S3, Supporting Information). Hence, similar to the retinal use case, the 3D devices exhibited once more the potential for multiple usage in acute settings.

An example of the recorded signals with the time point of insertion is shown in Figure 6B. To separate spiking activity of individual neurons from low‐frequency LFP signals, we filtered the raw signal into a high‐ and low‐frequency band (Figure 6B_i‐iii_). Immediately upon insertion we observed high‐amplitude spiking activity up to ≈200 µV while the low‐frequency activity was largely unperturbed. This shows that the pillars penetrated the cortex without notable perturbation of the neural tissue or ongoing activity patterns and can be used to efficiently record spiking activity from cortical neurons. Additional histological analysis with fluorescently labelled pillars confirmed that the pillars were successfully inserted into the cortex and reached the superficial cortical layers 2/3 (Figure S9, Supporting Information).

After retracting the wooden rod, the 3D MEA reliably remained in the tissue and simultaneously captured the spontaneous spiking activity from individual cortical neurons from several electrodes (Figure 6C_i_–C_iii_). Spike sorting revealed that each electrode captured spiking activity from several neurons at the same time, including multi‐unit recordings (Figure 6C_i_ orange spikes and Figure 6C_iii_ pink spikes) as well as potential individual neurons (green spikes in Figure 6C_i_–C_iii_). To also induce neural responses to sensory stimulation, we periodically stimulated the facial whiskers with two mild air puffs every 5 s and observed clear sensory responses to each whisker stimulus (Figure 6D_i_). Moreover, a repeated foot‐pinch induced even stronger bursts of action potentials that were time‐locked to sensory stimulation (Figure 6Dii).

These results clearly demonstrate that our 3D MEA design can be used to reliably record low‐frequency LFPs and spiking activity from cortical neurons while capturing functional responses to sensory stimulation. Although the background noise captured during the in vivo recordings exhibited a higher background noise than the retinal measurements, ± 20 µV versus ± 5–15 µV, respectively, the SNR of the in vivo signals allowed the recordings of large spike amplitudes. Such differences in the recordings can be explained due to setup differences, as the in vivo setup was not performed inside a Faraday cage and is therefore susceptible to interference noise coming from external sources, such as light sources or the heating pad that maintains warm the animal along the experiment. Furthermore, our devices comprise a design and hold electrochemical and mechanical properties (see Section 2.2) that are well‐suited to isolating neural activity from different cortical depths with single‐cell resolution, allowing the simultaneous recording of functional activity in different cortical locations and across cortical layers.

## 3 Conclusion and Outlook

In this work, we presented a novel, straightforward, versatile, and highly customizable 3D MEA fabrication process that yielded high‐aspect ratio 3D microelectrodes for the investigation of complex 3D neuronal systems. We use a 3D lithography tool based on 2PP to print hollow pillars onto different planar MEA substrates, using the pillars as templates to guide the electrochemical deposition of conductive materials, such as Au and PEDOT:PSS, to allow the growth of the 2D electrodes into the third dimension. The novelty of our process when compared to other approaches reported in the literature^[^ ^30^, ^31^ ^]^ relies on the idea of using hollow pillars instead of solid pillars. Hence, the hollow pillars produced in a single photolithography step serve the double purpose of directing the electrochemical deposition and passivating the conductive materials. Thereby, the fabrication steps to modify a planar MEA substrate into a 3D MEA are minimized, as only two post‐processing steps are needed to achieve a conductive pillar: printing of a hollow pillar, which serves as a template for the subsequent electrodeposition of the conductive material.

Likewise, our approach showed to be processing‐wise versatile and reproducible, as it was possible to fabricate 3D MEAs on both, stiff (SU‐8 on quartz) and flexible (PaC) MEA substrates, with different designs. Hence, the customizability of the 2PP printing together with the possibility of implementing the prints on different substrate materials, and technically with different electrodeposited conductive materials (e.g., Pt, PEDOT:PSS), makes this process easily adaptable to any electrode geometry and with the potential to be deployable on any planar MEA device. Thus, the electrochemically driven process to fabricate 3D microelectrodes could be also implemented as a post‐processing step to integrate, for example, 3D stimulating/recording microelectrodes with CMOS technology. In such a case, a CMOS‐compatible encapsulation and the use of thin film layers, such as titanium and tungsten, can be used in the planar chip to block the diffusion of metal contacts (e.g., Au or Pt) into silicon so that metal electrodes and via interconnects are possible,^[^ ^57^ ^]^ making the proposed process compatible with CMOS technology.

Although a maximum of 16 electrodes per 3D MEA were tested, the approach is scalable for the implementation of high‐density MEAs. Given that 2PP technology allows the printing of multiple pillars in one step and the electrochemical deposition process can be controlled individually and performed simultaneously for different pillars, different pillar heights and geometries can be implemented without increasing manufacturing time. Although 2PP lithography is not yet scalable for mass production capabilities, self‐aligned polymer‐based templates as proposed in this work could be also implemented using standard contact lithography and surface micromachining processes (e.g., spin coating, etch‐back processes, bonding techniques) by increasing production capability but sacrificing design customizability.

Beyond the straightforward fabrication process, another significant advantage of the introduced approach is the high degree of design and processing freedom to achieve, and surpass upon further optimization, high aspect‐ratio pillars of up to 33:1 with electrode diameters down to 8 µm and arbitrary heights of up to 400 µm in the same probe. Thus, we tailored 3D MEAs with form factors that have suitable electrochemical properties for electrophysiological recordings and that are also mechanically stable for their deployment in different neural targets.

Displaying its diverse utility, the technology exposed here enabled the recording of neural activity from in vitro neuronal cultures, acute neural slices, such as explanted retinas, and in vivo neural tissue, such as the brain cortex in rodents. As a result, our 3D MEA technology showed neural recordings with high SNRs (see Table S2, Supporting Information), the possibility to capture both, action potentials with single cell resolution, as well as population LFPs, for both spontaneous neural activity and physiological neural responses to sensory stimuli. Given the robust neural recording capabilities exposed and the different deployment modalities shown (in vitro and in vivo approaches), our 3D MEA technology can be applied to various other model systems beyond the use cases shown here, such as human brain slices, organoids, or invasive BCIs, therefore allowing many future applications for the study of neural network function. Even more, although in some applications the 3D MEAs are meant to be single use, the devices showed mechanical stability upon multiple insertions/retractions in acute applications, either in vitro or in vivo, thereby exhibiting potential re‐usability capabilities.

Lastly, due to the possibility of implementing this 3D MEA technology with polymeric materials with a low Young's modulus, such as PaC, and the low bending stiffness of individual pillars in our 3D MEAs, our devices show the potential to reduce the implantation footprint and foreign body reactions in comparison to other state‐of‐the‐art 3D neural probes used as prosthetic devices, such as stiff Utah arrays. While our 3D MEAs need further chronic testing to determine their long‐term stability and influence on foreign body reactions, the ease of fabrication and the customizability of our technology opens the door for the implementation of chronic applications in a variety range of 3D neuronal models, such as organoid electrophysiology tools for drug screening or personalized invasive BCIs for the restoration of lost sensorimotor functions (e.g., visual prostheses targeting the retina or the visual cortex). Our approach therefore facilitates the creation of customized designs that can be adapted to the specific needs of an application or in the future, of a patient, and enables the use of biocompatible materials that can facilitate the path of 3D MEA technology towards clinical translation.

## 4 Experimental Section

### 3D MEA Fabrication

The microfabrication processes were carried out at the Helmholtz Nano Facility^[^ ^58^ ^]^ and the laboratories of the Institute of Biological Information Processing‐3 (IBI‐3), Bioelectronics, at *Forschungszentrum* Jülich. Biocompatible materials such as parylene‐C,^[^ ^59^, ^60^ ^]^ SU‐8,^[^ ^61^ ^]^ IP‐L,^[^ ^32^, ^33^, ^34^ ^]^ Au,^[^ ^62^ ^]^ and PEDOT:PSS^[^ ^37^ ^]^ were used for the fabrication of the 3D MEAs. An overview of the microfabrication process flow of planar MEAs is given in Figure S10 (Supporting Information).

### Planar, Rigid MEA for In Vitro Applications

Stiff MEAs for in vitro applications comprise a quartz substrate, Ti/Au/Ti metal layer and an SU‐8 passivation layer, containing electrode openings of 10 µm or 6 µm in the passivation layer. From there, feedlines guide the current from the electrodes to the edges of the chip where the so‐called contact pads are located. These contact pads are non‐passivated metal structures which ensure the ability to contact the electrodes to external electronics. The dimension of the entire chip is 24×24 mm.

First, the quartz substrates were cleaned with acetone and IPA and then baked at 150°C for 5 min on a direct contact hot plate. To define the metal contact pads, feedlines, and electrodes the photoresist LOR 3B (MicroChemicals GmbH, Germany) was spin‐coated on the cleaned quartz substrate at 3000 rpm for 45 s with a ramp of 500 rpm s^−1^, followed by a soft‐bake at 150°C for 5 min on a direct contact hot plate. Afterwards, a second photoresist nLOF 2020 (MicroChemicals GmbH, Germany) is spin‐coated at 3000 rpm for 45 s with a ramp of 500 rpm s^−1^ and soft‐baked at 110°C for 1 min. This photoresist was then exposed at 20 mJcm^−^^2^ with broadband UV using a mask aligner (MA8/BA8, SÜSS MicroTec), followed by a post‐exposure bake at 110°C for 1 min, and a developing step in AZ 326 MIF (MicroChemicals GmbH, Germany) for 40 s. The substrates were then evaporated with a metal stack of 10/100/10 nm of Ti/Au/Ti using an electron‐beam assisted evaporation machine (Balzer PLS 570, Pfeiffer). The followed lift‐off process for nLOF2020 was done in acetone for 3 h. The substrates were then cleaned in acetone, IPA, and deionized water, and LOR3B was removed with AZ 326 MIF. To increase the adhesion of the passivation, the substrates were placed in a Piranha solution (H3O+/H2SO4 2:1) for 5 min. SU‐8 2002 (MicroChemicals GmbH, Germany) was then spin‐coated at 3000 rpm for 45 s with a ramp of 500 rpm s^−1^ and soft‐baked at 90°C for 1 min. To define the electrode and bond‐pad openings in the passivation, SU‐8 was exposed at 162 mJ cm^−^^2^ with UV light at 365 nm using a mask aligner (MJB4, SÜSS MicroTec) followed by a post‐exposure bake at 90°C for 1 min and a developing in MR Dev 600 (MicroChemicals GmbH, Germany) for 100 s and IPA for 20 s. To finalize the planar MEA fabrication the Ti layer on top of the Au layer at the electrode and bond pads was removed using reactive ion etching (RIE) with a gas mixture of Ar/O2 30/2, RF/ICP powers of 50/500 at 10°C for 15 s.

### Planar, Flexible MEA for Neural Implants

The fabrication of the planar MEA for in vitro and in vivo neural applications consisted of the deposition of two flexible thin film layers and one metal layer in between. First, a 5 µm thick PaC layer was deposited on a host silicon wafer via chemical vapor deposition using a PDS 2010 Labcoater 2 (Specialty Coating Systems Inc., USA). In contrast to the rigid MEAs, LNR003 was used to pattern the electrodes, feedlines, and contact‐pads for the metallization process. After dehydration of the PaC substrate at 150°C for 5 min, LNR003 was spin‐coated at 4000 rpm for 45 s with a ramp of 500 rpms^−1^ and soft‐baked at 120°C for 2 min. During the exposure with a maskless‐aligner (MLA150, Heidelberg Instruments, Germany) a dose of 320 mJ cm^−2^ and defoc of 2 were applied. The metallization process for a 20/100/10 nm thick stacked layer of Ti/Au/Ti was performed using electron‐beam assisted evaporation (Balzer PLS 570 Pfeiffer) and a lift‐off process using acetone. As a passivation layer, a second 5 µm‐thick PaC layer was then deposited as described before. The shape of the probes and the openings of the electrodes and contact pads were patterned in the next step. The positive photoresist AZ12XT was spin‐coated at 1000 rpm for 180 s with a ramp of 200 rpm s^−1^, soft‐baked at 110°C for 4 min, and exposed with the MLA150 using a dose of 350 mJ cm^−2^ and defoc of 2 followed by a baking step of 90°C for 60 s. To finalize the planar flexible MEA fabrication, the outline of the shape of the device, the contact pads, and electrode openings, PaC was etched with RIE using an O2/CF4 gas mixture. After the printing step, the flexible PaC probes were then released from the host Si‐wafer and flip‐chip bonded to customized printed‐circuit boards (PCBs) to connect them to the measurement system for electrophysiology.

### Printing of 3D Designs with 2PP

The 3D structures were designed using CAD software, exported as STL files, and converted to print job instructions using Describe (Software by NanoScribe GmbH). For the fabrication of a 3D device a 2PP 3D printer (Photonic Professional GT2, NanoScribe GmbH, Germany) was used. In this process an erbium‐doped femtosecond laser source (center wavelength 780 nm) was focused into a liquid droplet of a photo resin. The used photoresin was IP‐L 780 developed by the company NanoScribe for high‐resolution prints down to 200 nm. The biocompatibility of the IP‐L photoresin is confirmed by Nanoscribe and other customers.^[^ ^32^, ^33^, ^34^ ^]^ Two objectives, a Zeiss 25XNA0.8 and a Zeiss 63XNA1.4, were used to print the pillars, depending on the electrode layout. When using the 25X objective a scan speed of 50 000 µm s^−1^, a Laser Power of 100% and a Power Scaling of 1.2 were used. The slicing distance was set to 700 nm and the hatching distance to 400 nm. For the 63X objective a scan speed of 8000 µm s^−1^, a Laser Power of 100% and a Power Scaling of 1.0 was used. The slicing distance was 300 nm, and the hatching distance was 200 nm. As the optical properties of the printed polymer and the PaC/SU‐8 passivation is too similar, the interface between both cannot be found automatically by the 3D printer. Therefore, the interface must be found manually by adjusting the focus of the laser to ensure a high adhesion between the 3D print and the substrate. For developing the polymer after the printing, the samples were placed into Mr‐Dev 600 developer for 10 min followed by another 10 min in fresh Mr‐Dev 600 to ensure complete development. Finally, the sample was placed into IPA for another 5 min and then air dried.

To enhance the adhesion of the pillar to the planar MEA substrate, a 3 µm‐thick doughnut‐shaped base plate was added to the base of the hollow pillar to increase the contact area of the pillar. The contact area (*CA*) of a pillar can be calculated as follows:

(1) $$CA=\pi \cdot \left(R_{o}^{2}-R_{i}^{2}\right)$$

In Equation 1, *R_o_* and *R_i_* are the outer and inner radii of the printed structure, respectively. The bare hollow pillar has an inner/outer diameter of 8/12 µm, giving a wall thickness of 2 µm. Considering this, the contact area between the bare pillar and the substrate is 62.8 µm^2^. By adding a base plate of ≈50–100 µm diameter at the bottom end of the pillar, the contact area is then between 1913.23 – 7803.7 µm^2^.

### Template‐Assisted Electrodeposition of Conductive Layer

For the electrochemical deposition of Au, a liquid Au bath aqueous solution containing 50 mm AuCl_4_ was used. First, the surface was activated with oxygen plasma at a power of 100 W and pressure of 0.8 mbar for 1 min, enabling afterwards the influx of the Au solution into the hollow pillar. The Au deposition was then done in 2 steps (Figure 2B,C_i_). For the first step a constant potential (chronoamperometry) of −1.3 V was applied until the current reached a value of −100 nA. A second step was then used to create a homogeneous cap on top of the pillar with a slight and controlled overgrowing of the Au. For that a constant current of −100 nA was applied for 20 s. The deposition process was conducted using a multichannel potentiostat (CH Instruments Inc., USA) and a three‐electrode set up with an Ag/AgCl reference electrode and a Pt counter electrode.

During the deposition process, the following electrochemical reaction takes place:

(2) $${\mathrm{AuCl}}_{4\left(aq.\right)}^{-}+3e^{-}\to {\mathrm{Au}}_{\left(s\right)}+4{\mathrm{Cl}}_{4\left(aq.\right)}^{-}$$

To further improve the electrochemical properties, PEDOT:PSS was deposited from a EDOT:PSS solution containing 0.1 m of EDOT and 75 g mL^−1^ of PSS using again the multichannel potentiostat (CH Instruments Inc., USA) with a three‐electrode set up with an Ag/AgCl reference electrode and a Pt counter electrode. A cyclic voltammetry step was performed with the initial voltage set to 0 V, the final voltage set to 1 V and the scan rate was 0.1 V s^−1^ for 2 – 15 cycles.

### Electrochemical Characterization

Electrochemical impedance spectroscopy was done with a Biologic VSP‐300 (Bio‐Logic SAS, Claix, France) potentiostat using a configuration of three electrodes with the MEA electrodes as working electrode, a Pt wire as counter electrode, and an Ag/AgCl pellet as reference electrode. The impedance was measured in 0.1 m phosphate buffered saline (PBS).

The thermal noise or Johnson‐Nyquist noise of microelectrodes arises from the random motion of charge carriers within the electrode material, the solution, or at the electrode‐solution interface and depends on the real part Re(Z) of the complex impedance Z integrated over the recording frequency band (*df*).^[^ ^63^ ^]^ By measuring the impedance between *f_1_* = 300 Hz and *f_2_* = 3 kHz, the thermal noise of the 3D PEDOT:PSS coated electrodes was calculated as follows:

(3) $$V_{m}=\;{\left(4k_{b}T\int_{f_{1}}^{f_{2}}Re\left(Z\right)\;df\right)}^{\frac{1}{2}}$$

In Equation 3, the Boltzmann‐constant is *kb*, the absolute temperature *T* = 300 K, and *Re(Z)* is the real part of the impedance.

### Mechanical Stability of 3D Electrodes

*P_e_* was computed following Eulers’ equation for long slender columns by Equation 4:

(4) $$F=\frac{\pi^{2}EI}{{\left(KL\right)}^{2}}$$

where *E* corresponds to the Young's modulus, the second moment of inertia is *I*, the column effective length factor *K* = 0.7 for fixed‐pinned boundary condition, and *L* is the length of the pillar. The simulations were carried out with COMSOL Multiphysics solid mechanics module and a stationary study. For the material properties of IP‐L 780, values given by the manufacturer Nanoscribe were used (*E* = 4.73 GPa, Poisson's ratio = 0.3, and density = 1.17 g cm^−3^).

For a hollow pillar the momentum of inertia *I* was determined as follows:

(5) $$I=\frac{\pi }{4}\;\left(r_{o}^{4}-r_{i}^{4}\right)$$

where the outer radius *r_o_* = 6 µm and the inner radius *r*_i_ = 4 µm. For the boundary conditions, a fixed constraint at the pillar base and a pinned situation are assumed at the tip of the pillar where it is in contact with the tissue. To simulate the linear buckling, stress comprised by a load of 2 mN divided by the surface area is applied at the pillar tip in a negative *Z*‐direction. A parametric sweep was used to evaluate at which height the insertion force exceeds the critical buckling load of the pillar.

### Imaging

After the fabrication of the planar MEA probes, pictures were taken with a Nikon L200N microscope. Pictures of the 3D printed structures were then taken using a SEM (Gemini 1550 instrument (Leo/Zeiss)). To improve the SEM investigations with enhancing the conductivity, a thin layer of iridium oxide (6 nm) was sputtered onto the sample (current 15 mA for 1 min). The imaging was taken at 3 kV acceleration voltage.

To cut through the pillars using a FIB first, a 400 nm thick layer of platinum was deposited using an electron beam‐induced deposition process. The sample was then tilted by 50°. A gallium ion beam of 0.2 nA was used to cut the pillar. Finally, a polishing step at 30 kV and 0.08 nA was performed.

### Cell Culture and Tissue Preparation for In Vitro Recordings

The use of primary tissues in this work has been approved by the *Landesumweltamt für Natur, Umwelt und Verbraucherschutz Nordrhein‐Westfalen*, Recklinghausen, Germany, under permit number 81‐02.04.2018.A, and has been conducted according to German animal protection law and ethics and is reported according to the ARRIVE guidelines.

### Neuronal Cell Culture

An oxygen plasma surface activation step was performed for 1 min which renders the surface of the 3D MEA to be hydrophilic. The power was set to 100 W, the pressure was set to 0.8 mbar. Prior to cell seeding, the chips were sterilized in 70% ethanol and washed three times with sterile MilliQ water. Afterward, the chips were coated with 10 µg mL^−1^ poly‐L‐lysine (PLL, Sigma‐Aldrich, Steinheim, Germany) diluted in Hank's balanced salt solution (HBSS) 1/100 at room temperature and in sterile environment for one hour. Before seeding, the remaining coating solution was aspirated, and the chips were cleaned three times with HBSS. The cortical neurons were extracted from the hippocampus of Wistar E18 rat embryos and separated into individual cells by incubation at 37°C with 0.05% trypsin EDTA (Life Technologies GmbH, Darmstadt, Germany), 5% CO2, and 95% humidity for 10 min. The tissue was gently removed and washed 5 times with Supplemented Neurobasal medium (NB medium, Life Technologies GmbH, Darmstadt, Germany), 1% B‐27 supplement (Life Technologies GmbH, Darmstadt, Germany), 0.5 mM L‐glutamine (Life Technologies GmbH, Darmstadt, Germany) and 50 µg mL^−1^ of gentamicin (Sigma‐Aldrich, Steinheim, Germany). After the last washing step, the supernatant was replaced with fresh supplemented Neurobasal medium and the tissue was triturated until completely dissociated. Cells were counted with Neubauer improved cell counting chamber and the wanted number of cells was plated onto each sample. The samples were kept at 37°C and 5% CO_2_. One to four hours after cell seeding, the medium was replaced completely with medium. From the first day in vitro, every three to four days, half of the medium was replaced with fresh warm medium. After the experiments the samples were cleaned. Trypsin EDTA was used to remove the attached cells from the sample. For that, the sample was immersed into trypsin on a hot plate. After 20 min the trypsin was removed, and fresh trypsin was added for another 1 h to ensure proper detachment of the cells from of the complex 3D structures. Samples were then immersed in a 1% Tergazyme solution in ultra‐pure water for several hours.

### Cell Fixation and Imaging

To investigate the neuronal growth on the 3D printed structures via SEM, the neurons must be fixed. For that, the sample was rinsed three times with PBS (37°C, Sigma Aldrich, USA) and chemically fixed with 2% glutaraldehyde (GA) (Sigma Aldrich, USA) solution in PBS for 15 min at room temperature. Afterward, the sample was washed three times with PBS and MilliQ water to remove fixative residues. For SEM investigations the fixed neurons need to be dried. CPD was used for this purpose. First, water in the sample was moderately replaced with ethanol (intermediate medium). The sample was then incubated for 5 min in 10%, 30% and 50% ethanol, 15 min in 70% ethanol, followed by three times for 5 min in 90% and 95% ethanol. The sample was then transferred in 100% ethanol at 4°C and transported to the chamber of the CPD (CPD 030, BAL‐TEC Company). The chamber of the CPD was filled with 100% ethanol and the samples were placed inside. The chamber was cooled down to 10°C and ethanol was exchanged by CO_2_. The exchange process was repeated several times until the chamber was filled with CO_2_ followed by an increase in temperature to 40°C and in pressure to 73 bar. Eventually the chamber was evacuated while the sample was dried and dehydrated.

### Retina Explants

Light adapted retinas were explanted from Wistar rats according to the German animal protection law. Animals were first deeply anesthetized with CO_2_ and decapitated. Right away, the eyeballs were enucleated and immersed in fresh artificial cerebrospinal fluid (ACSF), which contained (in mm) 124 NaCl, 24 NaHC0_3_, 3 KCl, 1.25 NaH_2_P0_4_, 1.25 MgCl_2_, 2 CaCI, and 10 glucoses. The medium was constantly oxygenated with carbogen gas containing 95% O2 and 5% CO2 (The Linde Group, Germany) and pH of 7.4 was adjusted with sodium bicarbonate (NaHC0_3_). The preparation of the light‐adapted retinas was performed as reported before.^[^ ^52^ ^]^ One eyeball was opened along the *ora serrata* to extract the cornea, lens, and to carefully remove the vitreous body. The procedure was then executed with the second eye to ensure constant oxygenation. After that, the posterior eyeball was cut in half and the two pieces of retina were carefully isolated. One piece was then stored again in oxygenated ACSF and the other one was prepared for the first experiment. Here, the retina was placed on a donut‐shaped piece of filter paper with the ganglion cell layer facing downwards. It was then flipped (ganglion cells now facing upwards), placed inside the perfusion chamber, where it was fixed with insect pins to hold it in place.

### In Vivo Animal Testing

Animal experiments in this work have been approved by the *Landesumweltamt für Natur, Umwelt und Verbraucherschutz Nordrhein‐Westfalen*, Recklinghausen, Germany, under permit number 81‐02.04.2021.A021. Animal experiments have been conducted according to German animal protection law and ethics and is reported according to the ARRIVE guidelines. Mice for in vivo trials were breed and housed at the Institute of Biology 2 at RWTH Aachen. Mice were placed in a stereotactic frame and anesthetized using 1–5% of isoflurane. The skin at the skull was gently cut and pulled to the side after an initial incision. A craniotomy of about 4 mm in diameter was conducted at the left hemisphere using a biopsy punch and an orthopaedic drill (Eickemeyer). The surface of the dura was then cleared and subsequently covered with PBS. After an initial incision, the dura was then carefully removed with a hooked needle to gain access to the somatosensory cortex. The 3D flexible microelectrode was placed on top of the dried cortex using a 3‐axis micromanipulator (MTM‐3, World Precision Instruments). A wooden rod was fixed on a second micromanipulator (uMp4, Sensapex) and used to push the implant into the tissue. The insertion was done with step sizes between 100–250 µm and a velocity of 4000 µm s^−1^. For stable and low‐noise signal recordings, a reference pin was placed on the cerebellum.

### Histological Preparations

The pillars of the 3D MEAs were painted with a fluorescent infrared dye (DiD V22887, ThermoFisher) dissolved in ethanol. A 10 µL drop of the DiD solution was placed on the implant, leaving a thin layer of DiD on all pillars after the ethanol was fully evaporated. After inserting the implant, the pillars remained in the tissue for ≈30 min.

After the experiment, the mice were perfused with PBS and subsequently with 4% Paraformaldehyde (PFA) solution. The brain was explanted and kept in 4% PFA at 6°C. After one day, the brain was placed in 15% sucrose solution and after one more day moved to 30% sucrose solution. After the brain sunk to the bottom of the sucrose solution, it was frozen and cut into 75 µm‐thin slices using a commercial Cryotome (CM3050, Leica). The slices were then mounted on cover slides and fixed with mounting medium, containing a DAPI stain (Fluoromount, Thermofisher). The cover slides were then sealed with nail polish, and we used a commercial confocal microscope to obtain images of the cortical slices and the pillar implantation sites.

### Data Acquisition

The BioMAS, an in‐house amplification system,^[^ ^61^ ^]^ was used for in vitro recordings with stiff MEA chips with a built‐in 64‐channel headstage to perform electrical recordings. The sampling rate was set to 20 kHz and an Ag/AgCl pellet (World Precision Instruments) was used as reference electrode.

For in vitro and in vivo tissue recordings with flexible MEAs, the ME2100‐System (Multi Channel Systems MCS GmbH, Germany) and a 32 channel headstage (ME2100‐HS32‐M‐3m) were used. The NeuroNexus adapter (ADPT‐NN‐16/32) served to connect the headstage to our customized 16 channel PCBs. The headstage was mounted on a micromanipulator (Luigs & Neumann GmbH, Germany) to enable remotely controlled insertions. The McsMatlabDataTools Matlab toolbox (Multi Channel Systems MCS GmbH, Germany)^[^^]^ was used to import HDF5 files created by the ME2100‐System.

### Statistical Analysis

To evaluate the fabrication yield, first a successful print was defined as all pillars standing and aligned to the electrode openings. Then, the ratio of successful prints versus failed prints was determined (85%, N = 111). If a print was successful, the 3D MEA pillars were filled with Au and coated with PEDOT:PSS via electrodeposition. In a second evaluation step, the yield of the electrodeposition was evaluated using light microscopy pictures. First, ratios of filled versus unfilled pillars (55%, N = 51), and then, ratios of successful coating versus unsuccessful coating for filled pillars were computed (90%, N = 51).

For the electrophysiological recordings, offline data processing was performed using self‐written MATLAB (Mathworks Inc., United States) scripts. The spiking activity and LFPs were extracted from 6th order zero‐phased Butterworth filters using a bandpass with cut‐off frequencies of 100 Hz and 3 kHz, and a low pass with cut‐off frequency of 100 Hz. Spike sorting for each electrode was done with the help of the ultraMegaSort2000 algorithm.^[^ ^66^ ^]^ For SNR calculations, the spike amplitudes were extracted and divided by the standard deviation of the noise using time windows of 1 s. Means and standard deviations were computed for each recording type.

## Conflict of Interest

*Forschungszentrum* Jülich has filed a patent that covers the 3D MEA fabrication exposed in this manuscript, listing J.A.S., M.J., L.K., V.R.M, and A.O. as inventors.

## Author Contributions

J.A.S. and M.J. contributed equally to this work. J.A.S., M.J., V.R.M., and A.O. planned the study. J.A.S. and A.O. conceived the development of stiff 3D printed MEAs, and J.A.S., M.J., A.O, and V.R.M. conceived the development of flexible 3D printed MEAs. J.A.S., M.J., and S.D. fabricated the devices with the support of L.K., A.O., and V.R.M. J.A.S. characterized the probes with the support of M.J. and S.D. Cell culture experiments were carried out by J.A.S with the support of A.O. Retina experiments were conducted by M.J. and J.A.S. with the support of V.R.M. in vivo experiments were performed by S.M. with the support of M.J., S.D., L.K., J.A.S., and V.R.M. Simulations were carried out by M.J. with the support of J.A.S. and V.R.M. Data and figures were processed by M.J. with the support of J.A.S., S.M., A.O., and V.R.M. M.J., J.A.S., S.M., V.R.M., and A.O. wrote the initial draft of the manuscript. All authors reviewed and edited the manuscript. V.R.M and A.O. co‐supervised the study.

## Supporting information

Supporting Information

Supplemental Video 1

Supplemental Video 2

Supplemental Video 3

## Data Availability

The data that support the findings of this study are available from the corresponding author upon reasonable request.
