# Supplementary material for: Highly Customizable 3D Microelectrode Arrays for In Vitro and In Vivo Neuronal Tissue Recordings
Source: Adv Sci (Weinh). 2024 Jan 19;11(13):2305944. doi: 10.1002/advs.202305944 (PMC10987114; doi:10.1002/advs.202305944)
Supplement: Supplementary file 1 — Supporting Information [file ADVS-11-2305944-s001.pdf]

ADVANCED SCIENCE
Open Access

Supporting Information

for *Adv. Sci.*, DOI 10.1002/advs.202305944

Highly Customizable 3D Microelectrode Arrays for In Vitro and In Vivo Neuronal Tissue Recordings

J. Abu Shihada, M. Jung, S. Decke, L. Koschinski, S. Musall, V. Rincón Montes and A. Offenhäusser**

Supplementary Information

A Fully Customizable 3D Microelectrode Platform for In Vitro and In Vivo Neuronal Tissue Recordings

J. Abu Shihada[1,2,#], M. Jung[1,2,#], S. Decke[1], L. Koschinski[1,2,4], S. Musall[1,3], V. Rincón Montes[1*] and A. Offenhäusser[1*]

[1]Institute of Biological Information Processing (IBI-3) – Bioelectronics, Forschungszentrum, Jülich, Germany

[2]RWTH Aachen University, Aachen, Germany

[3]Helmholtz Nano Facility (HNF), Forschungszentrum Jülich, Germany

[4]University of Bonn, Faculty of Medicine, Institute of Experimental Epileptology and Cognition Research, Germany

[5]University Hospital Bonn, Germany

[#]These authors have equally contributed to this work

[*]Corresponding authors: v.rincon.montes@fz-juelich.de and a.offenhaeusser@fz-juelich.de

Supplementary Figures and Tables

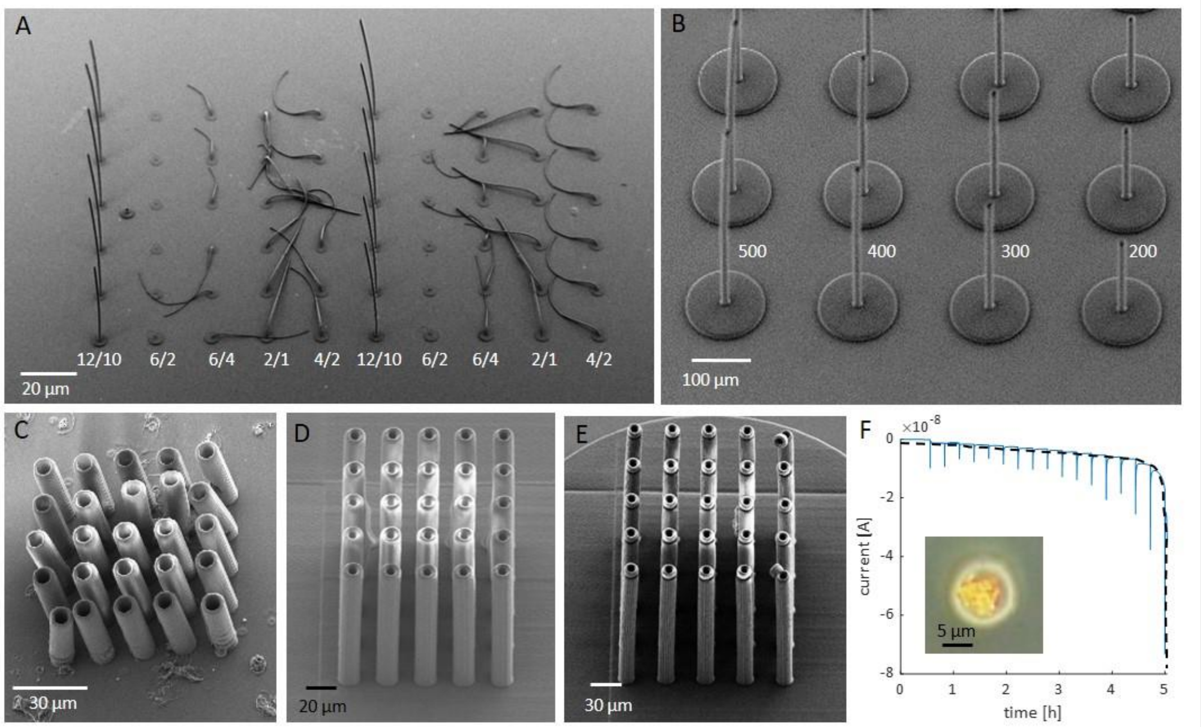

Figure S1. SEM pictures of tested configurations and limitations on a parylene-C substrate. A) Outer and inner diameter test parameters. From left to right 12/10, 6/2, 6/4, 2/1, 4/2, 12/10, 6/2, 6/4, 2/1, 4/2, where the first number is the outer and the second is the inner diameter in µm. The pitch of the pillars is 200 µm. B) Testing height parameters for straight pillars. From left to right: 500, 400, 300, and 200 µm high pillars with an outer diameter of 12 µm, an inner diameter of 8, and a pitch of 200 µm. C) Array of 50 µm high pillars with 20 µm pitch. D) Array of 150 µm high pillars with 25 µm pitch. E) Array of 200 µm high pillars with 35 µm pitch. F) Current-time curve of the electrochemical deposition of Au for a 400 µm high pillar.

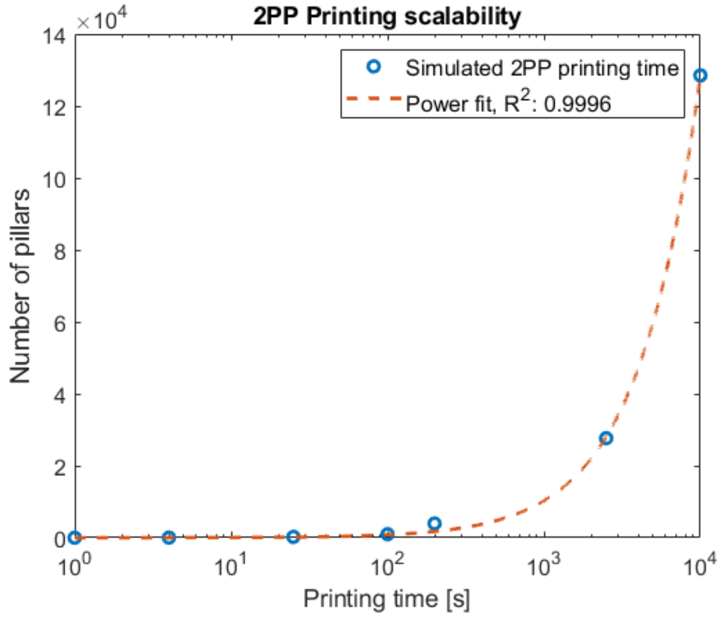

Figure S2. Scalability of two-photon polymerization (2PP) printing of hollow-pillars. 2PP printing time was simulated in DeScribe (Nanoscribe GmbH & Co. KG) considering a pillar length of 70 µm, an outer diameter of 12 µm, an inner diameter of 8 µm, a base plate of 100 µm in diameter, and a pillar pitch of 100 µm using a 25x objective lens. Block splitting was used when multiple pillars did not fit inside the printing voxel of 285 x 285 x 300 µm^3.

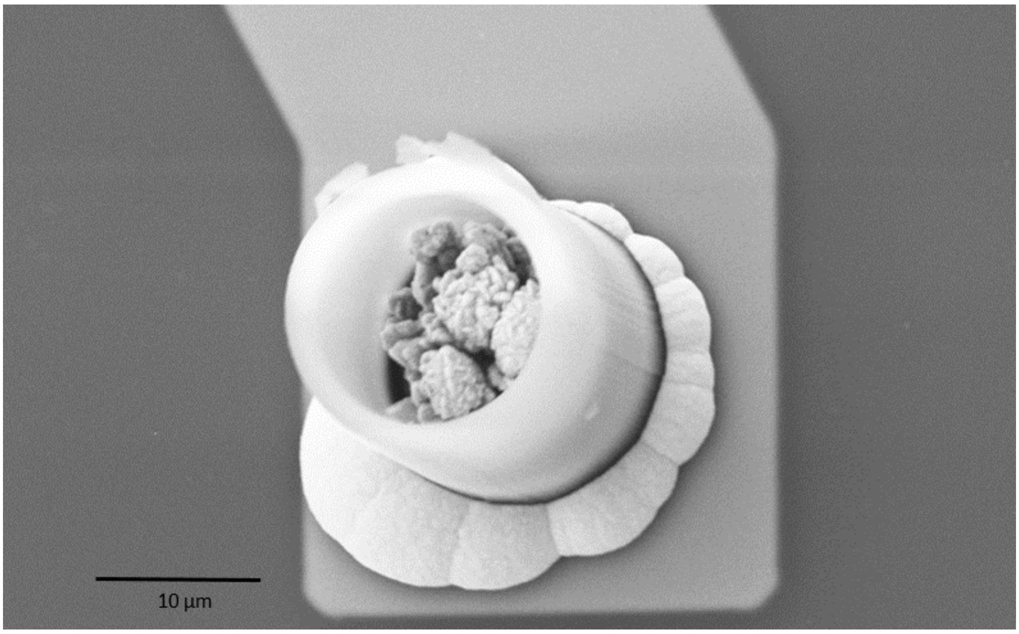

Figure S3. Challenges of template-assisted electrochemical deposition of Au. Overgrowth of Au at the base due to the poor adhesion of the 2PP-based hollow polymeric pillar during Au electrodeposition (private communication).

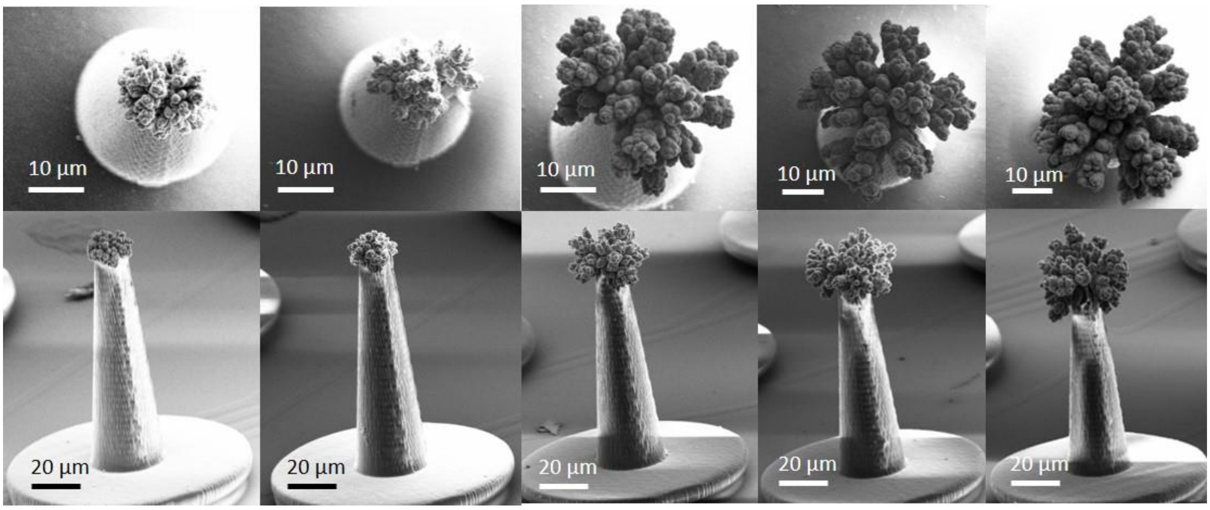

Figure S4. Characterization of PEDOT:PSS caps. PEDOT:PSS growth upon electrochemical deposition *via* cyclic voltammetry depends on the number of cycles (cycles C = 2 to 10).

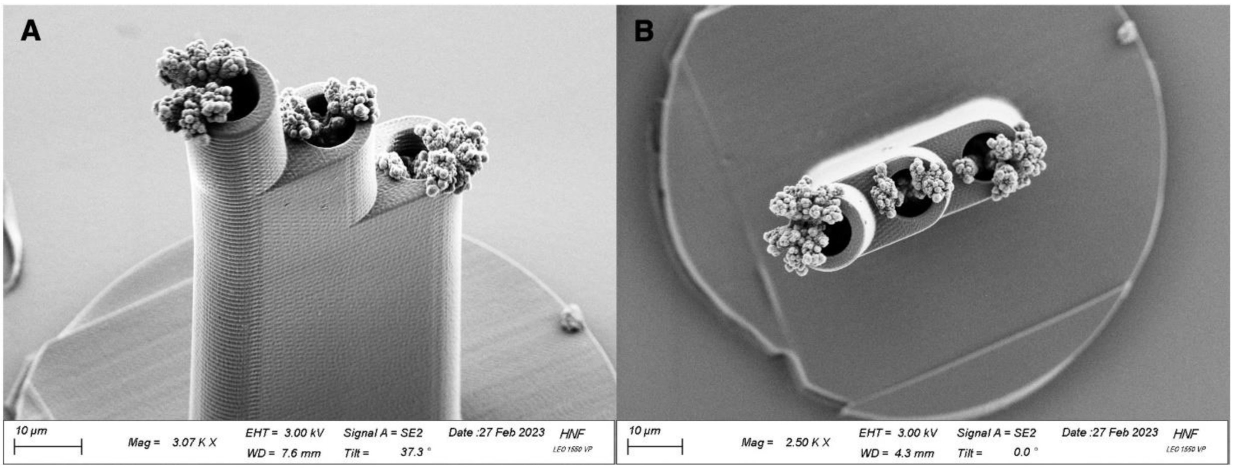

Figure S5. SEM pictures of a multisite 3D MEA with three electrodes coated with Au and PEDOT:PSS. A) Side-view of a probe with 80, 100 and 120 µm pillar heights with an opening of 8 µm, B) Top-view.

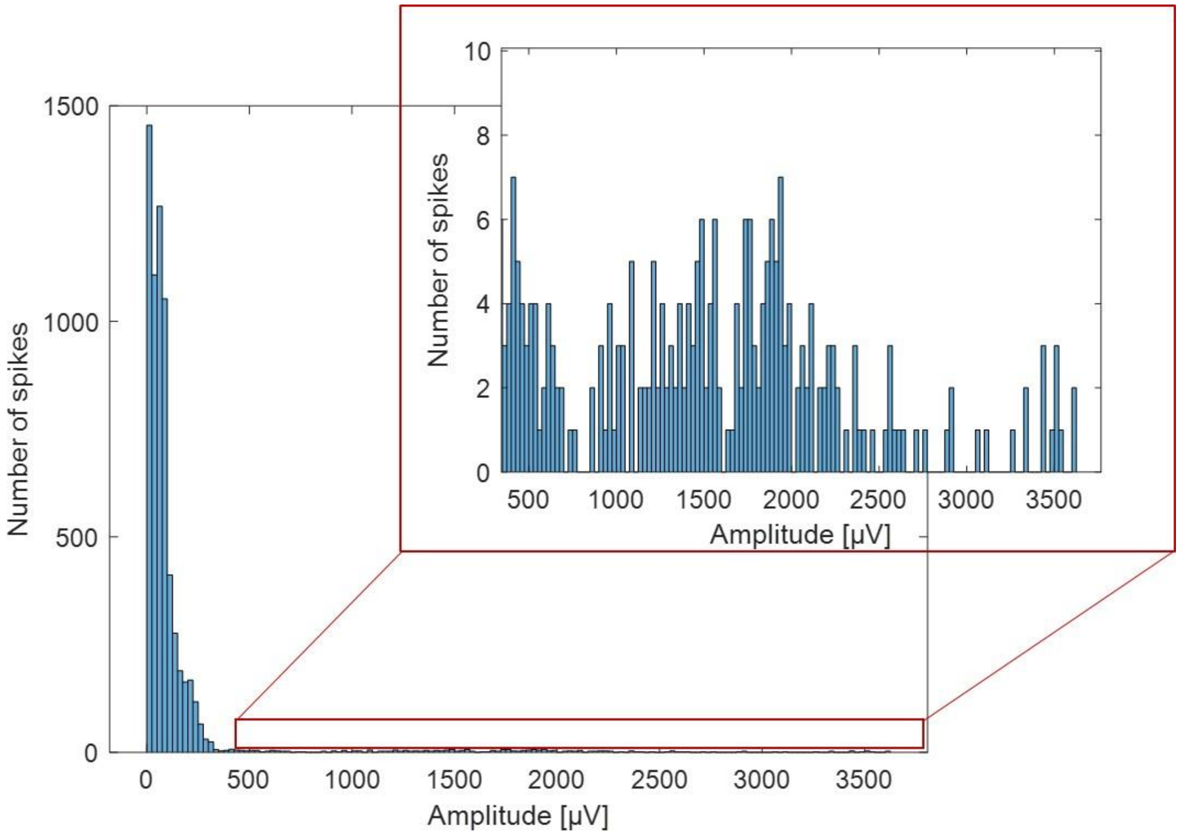

Figure S6. Histogram of the amplitudes of spikes from 3D neuronal cell culture recordings. The histogram shows the number of spikes sorted by their amplitude with a bin size of 25 µV. Spikes were captured by 28 recording electrodes.

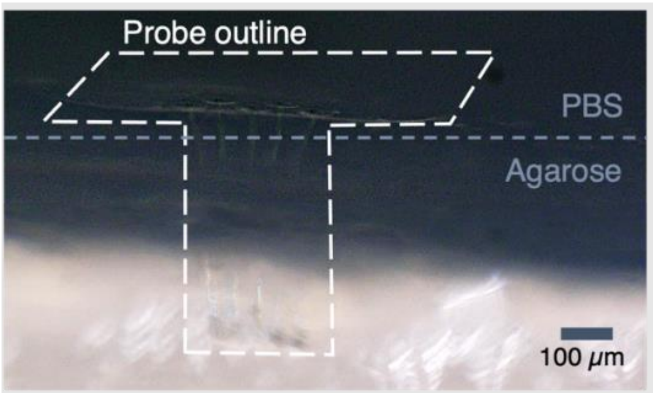

Figure S7. Insertion test of a flexible probe into an agarose gel. A flexible 3D-MEA with 16 pillars of 350 µm height was successfully inserted into agarose gel using a wooden rod to push the implant down with a step size of 250 µm and a speed of 4000 $µms^{-1}$. After the retraction of the wooden rod, the implant remained in its place and the individual pillars are still visible.

Table S1. Literature comparison of 3D electrode arrays.

Device	Components	Main Material	E-modul [GPa]	Pillar Length [µm]	Diameter (at the base) [µm]	E·I [Nµm²]	cross-section at centroid [µm²]	Reference
**This work**	Hollow IPL pillars filled with Au	IPL	4.73	Up to 500	straight pillars: 12<br>cone-shaped pillars: 35<br>multisite pillars: 36 x 12	3.86<br>50.73<br>11.59	110<br>541<br>250	This work
**Silicon microneedle array**	Needle electrodes	Si	160	100	10	78.5	78.5	Lee et al. 2022
**Inkjet-Printed and electroplate electrodes**	Solid pillar electrodes	AgNP ink	82	560	32	4220	804	Grob et al. 2021
**3D printed electrodes**	Cone-shaped pillar electrodes	ormocomp	1	350	50	97	1105	Brown et al. 2022
**Utah Array**	Cone-shaped needle electrodes	Si	160	1500	90	163040	3579	Campbell et al., 1991
**Sea of Electrodes**	Cone-shaped needle electrodes	Si	160	1200	20	398	177	Zardini et al, 2021
**Michigan-Style Flexible probes**	Flat shanks	PaC	2.76	5500	20 x 110<br>(thickness x width)	6122	2200	Wang et al., 2020
**Michigan Array**	Flat shanks	Si	160		10 x 50<br>(thickness x width)	16667	500	Wise at al., 1970

The bending stiffness was computed by the product of the Young's modulus *E* and the second moment of inertia *I*:

$$E \cdot I$$

Here, *I* depends on the geometry of the respective electrodes and was calculated as follows for solid pillars with an outer radius *r*

$$I = \frac{\pi}{4} \cdot r^4.$$

For solid cone-shaped pillars, *r* was taken at the centroid of the cone at ¼ of the height, leading to $r = \frac{3}{4} r_b$ with the radius r_b at the base of the pillar. For hollow pillars with an outer radius r_o and inner radius r_i,

$$I = \frac{\pi}{4} \cdot (r_o^4 - r_i^4)$$

For multisite pillars assuming a cross-section of 3 hollow pillars, I was calculated by

$$I = 3 \cdot \frac{\pi}{4} \cdot (r_o^4 - r_i^4)$$

respectively.

For planar shanks with a rectangular cross-section with width b and thickness d,

$$I = \frac{db^3}{12}$$

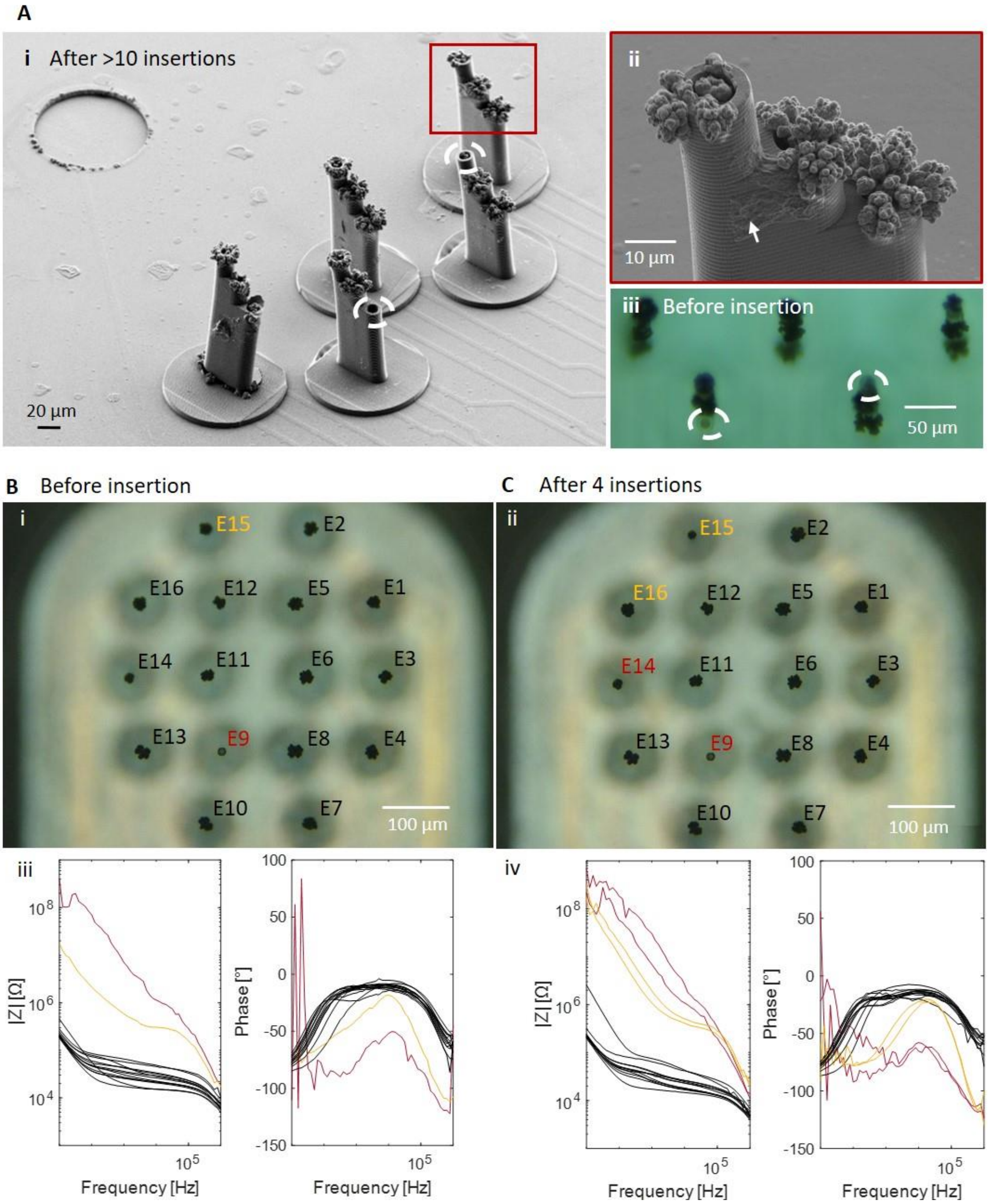

Figure S8. Inspection of 3D probes after several insertions into retinal tissue. A) Multisite probe with five shanks comprised of three electrodes each with heights of 80, 100, and 120 µm after it was inserted >10 times

into retinal tissue (A_i). Retinal tissue residues are visible (white arrow) in the zoomed-in picture (A_{ii}). (A_{iii}) shows a top view of the probe with a light microscope picture before the insertion. Here, two pillars were not filled with Au (marked with white dashed circles). B) Light microscope pictures of an 80 µm high probe with 106 µm pitch between the pillars before (B_i) and after (B_{ii}) four insertions into retinal tissue. Impedance values were measured before and after insertion. Here, the pillars of E14, E15, and E16 presented an increased impedance, however, upon optical inspection, both, the 3D printed pillars and the electrode coatings of E16 and E14 remained intact leading to the conclusion that either the feedlines of the flexible cable broke or the Au-string inside the pillar was affected.

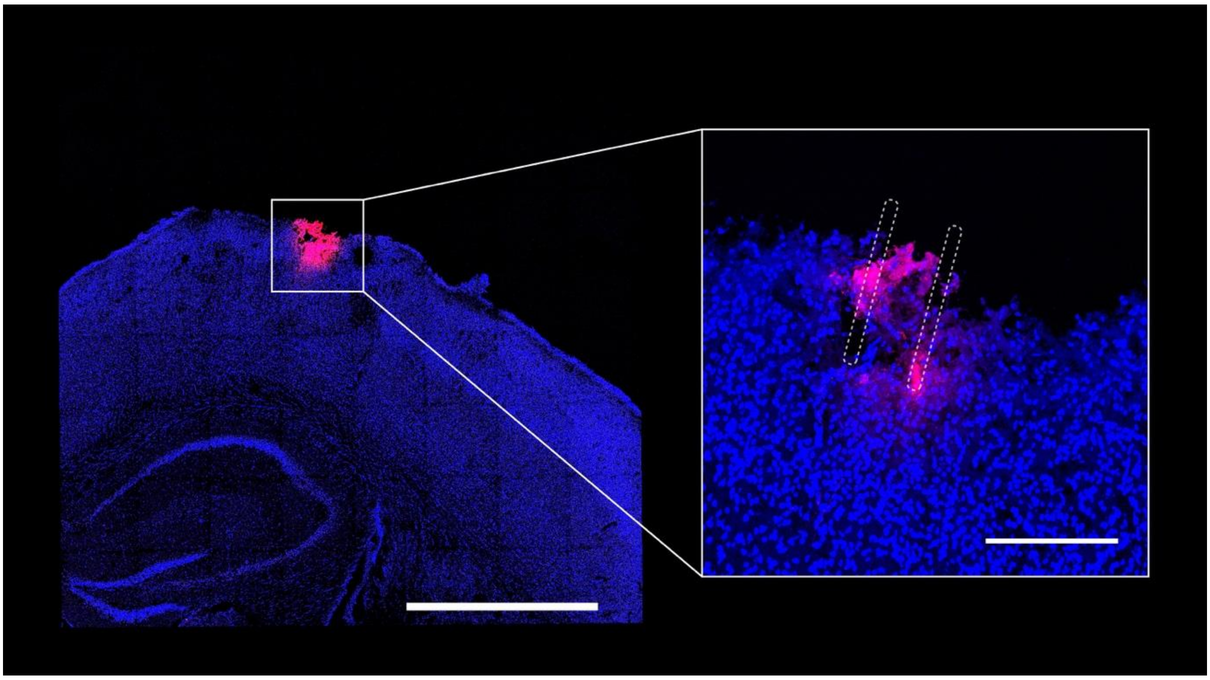

Figure S9. Example brain slices with electrode insertions. The left image shows an example brain slice from an implanted mouse with fluorescently labeled electrode tracts in the somatosensory cortex. The blue channel shows fluorescence due to DAPI-staining of all cell nuclei to visualize different brain structures. The red channel is a false-color image of infrared fluorescence due to coating the electrode pillars with the fluorescent dye DiD. The right image shows a magnification of the implanted brain region with fluorescence from two electrode tracts (indicated by dashed outlines). Scale bar is 1 mm on the left and 0.2 mm on the right image.

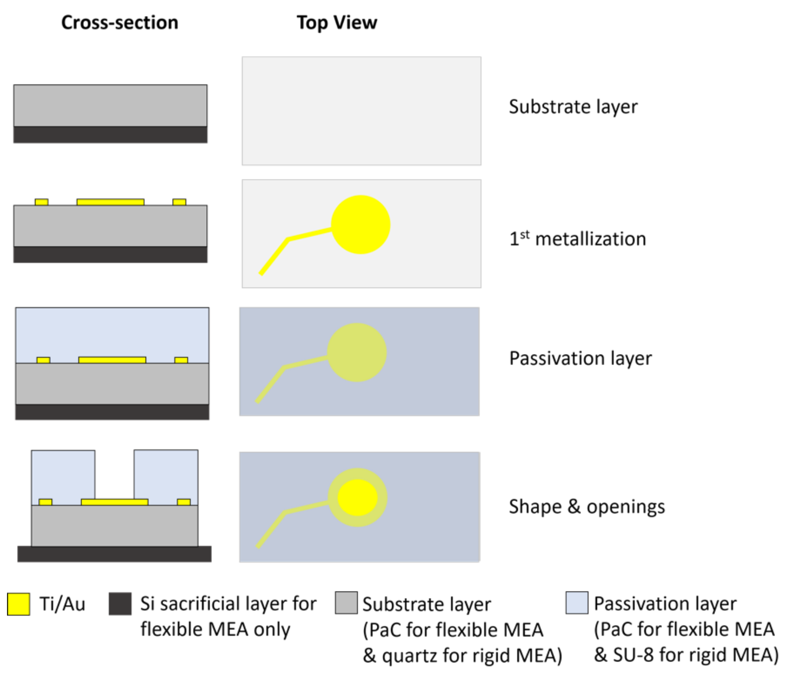

Figure S10. Fabrication of rigid and flexible planar MEA before 3D MEA fabrication. The process flow consists of a first chemical vapor deposition (CVD) of parylene-C (PaC) on a silicon (Si) host wafer for a flexible MEA while the substrate layer for rigid MEA is quartz (no Si wafer used). Then, metallization by E-beam evaporation of a Ti/Au/Ti layer stack, followed by a 2nd PaC for flexible or SU-8 for rigid MEA respectively. Lastly, the shape and electrodes are opened by reactive-ion-etching. In the case of the stiff planar MEA, electrode and contact pad openings were achieved by the photopatterning of SU-8.

Table S2. Overview of signal-to-noise ratio across different use cases using 3D MEAs in *in vitro* and *in vivo* applications. SNRs are given for the recordings shown in the Figures of the main manuscript.

**Use case**	**SNR**	**#Spikes (*N*)**
In vitro 3D neuronal cell culture	11.8 ± 5.6	6,482
In vitro retina approach	7.3 ± 2.1	675
In vitro retina with *in vivo* approach	5.5 ± 2.8	194
In vitro retina using multisite probe with *in vivo* approach	9.8 ± 6.2	1,726
In vivo mouse cortex	6.9 ± 2	10,068

Supplementary videos

Supplementary video SV1: 3D MEA insertion into agarose. Insertion into agarose gel using a multisite probe with 5 shanks containing 3 electrodes each with heights of 460, 480, and 500 µm. The probe was gently pushed inside the geld using a wooden rod fixated to a micromanipulator.

Supplementary video SV2: 3D MEA *in vivo* insertion. Insertion into a mouse cortex after removing the dura under in-vivo conditions. A probe with cone-shaped pillars with a height of 200 µm and a pitch of 106 µm was used. The probe was gently pushed inside the gel using a wooden rod fixated to a micromanipulator.

Supplementary video SV3: 3D MEA *in vivo* retraction. *In vivo* probe retraction (same probe from SV2) after performing electrophysiological recordings.
